# Effects of alcohol on the transcriptome, methylome and metabolome of *in vitro* gastrulating human embryonic cells

**DOI:** 10.1242/dmm.052150

**Published:** 2025-06-18

**Authors:** E. Wallén, K. Rämö, J. Vehviläinen, J. Sokka, M. Lehtonen, T. Otonkoski, R. Trokovic, P. Auvinen, O. Kärkkäinen, N. Kaminen-Ahola

**Affiliations:** ^1^ Environmental Epigenetics Laboratory, Department of Medical and Clinical Genetics, Medicum, Faculty of Medicine, University of Helsinki, 00290 Helsinki, Finland; ^2^ Research Programs Unit, Stem Cells and Metabolism and Biomedicum Stem Cell Centre, Faculty of Medicine, University of Helsinki, 00290 Helsinki, Finland; ^3^ School of Pharmacy, Faculty of Health Sciences, University of Eastern Finland, 70211 Kuopio, Finland; ^4^ Department of Pediatrics, Children's Hospital, Helsinki University Central Hospital, University of Helsinki, 00290 Helsinki, Finland

**Keywords:** Prenatal alcohol exposure, FASD, Gastrulation, DNA methylation, Embryonic development, Endoderm, Mesoderm, Ectoderm

## Abstract

Prenatal alcohol exposure (PAE) affects embryonic development, causing a variable fetal alcohol spectrum disorder (FASD) phenotype with neurodevelopmental disorders and birth defects. To explore the effects of PAE on gastrulation, we used an *in vitro* model with subchronic moderate (20 mM) and severe (70 mM) ethanol exposures during the differentiation of human embryonic stem cells into germ layer cells. We analyzed genome-wide gene expression (mRNA sequencing), DNA methylation (EPIC Illumina microarrays) and metabolome (non-targeted LC-MS) of the endodermal, mesodermal and ectodermal cells. The largest number of ethanol-induced alterations were observed in endodermal cells, whereas the most prominent changes were in ectodermal cells. Methionine metabolism and genes of the main signaling pathways involved in gastrulation and body patterning were affected by ethanol in all germ layers. Many of the altered genes, including *BMP4*, *FGF8*, *SIX3* and *LHX2*, have previously been associated with PAE and phenotypes of FASD, like defects in heart and corpus callosum development as well as holoprosencephaly. Our findings support the early origin of alcohol-induced developmental disorders and strengthen the role of methionine cycle in the etiology of FASD.

## Teaser

**Summary:** Alcohol exposure affects methionine metabolism and the regulation of developmentally essential genes in an *in vitro* model of human gastrulation.

## INTRODUCTION

Prenatal alcohol exposure (PAE) is one of the most significant causes of developmental disability affecting 3–5% of individuals in the Western world (Roozen et al., 2016). PAE can cause a wide range of developmental defects including neurodevelopmental disorders (NDDs), structural malformations and growth disturbances referred to as fetal alcohol spectrum disorders (FASD). Its most severe form is fetal alcohol syndrome, which is characterized by craniofacial malformations, central nervous system defects as well as prenatal and postnatal growth restriction (Hoyme et al., 2016).

Many teratogenic effects of alcohol on the developing embryo have been traceable back to gastrulation (Goodlett and Horn, 2001; Lipinski et al., 2012), resulting in a high incidence of FASD (Sulik, 1984; Maier and West, 2001; Guerri, 2002). Occurring in the third week post fertilization in human, gastrulation is one of the most critical events in development, establishing directionality within the developing embryo and priming the system for organogenesis. During gastrulation, embryonic stem cells differentiate into three embryonic germ layers. The endoderm, the innermost layer of the embryo, forms the linings of the gastrointestinal, respiratory and urinary tracts. The middle layer, the mesoderm, forms the connective tissue, smooth muscle, cardiovascular system, skeleton, blood and reproductive systems. The outermost layer is the ectoderm, from which the external ectoderm as well as the neuroectoderm, including spinal cord, neural crest and neural tube-brain, develop. Alcohol-induced changes in the ectodermal cells are of particular interest, as the developing nervous system is known to be especially sensitive to the effects of PAE (Guerri, 2002), and alcohol-related NDDs appear to be the most common diagnosis among the FASD (Wozniak et al., 2019). However, due to the technical and ethical limitations, the effects of alcohol exposure on the embryonic germ layers in human are mostly unknown.

The spatiotemporal control of gene expression is essential for directing cell type specification, migration and localization. DNA methylation (DNAm) is a covalent epigenetic modification on the DNA strand that, by affecting chromatin density and the binding of transcription factors, regulates gene expression according to the cell type and developmental stage. Early pregnancy, a period of dynamic cell divisions, DNA replication and establishment of cell type-specific epigenetic profiles during epigenetic reprogramming, is particularly sensitive to environmentally induced epigenetic alterations (Susiarjo et al., 2013; Tobi et al., 2015). The establishment of DNAm profiles and their maintenance in mitotic cell divisions are accomplished by DNA methyltransferase enzymes. Early alcohol-induced DNAm alterations have been observed in human and mouse embryonic stem cells (hESCs and mESCs, respectively) (Sánchez-Alvarez et al., 2013; Khalid et al., 2014; Auvinen et al., 2022) as well as in the offspring of the early PAE mouse model (Kaminen-Ahola et al., 2010; Zhang et al., 2015; Bestry et al., 2024). As a membrane-permeable molecule, alcohol can affect DNAm during embryonic and extraembryonic development even before implantation, as mouse studies have shown (Legault et al., 2021, 2024). It has been hypothesized that, beyond immediate cell death or DNA strand breaks, PAE may impact the methylation potential of the cell and, consequently, alter the sensitive period of epigenetic reprogramming. Previous studies have suggested that alcohol can affect methylation capacity through methionine metabolism (Kruman and Fowler, 2014; Ngai et al., 2015) and by interfering with the activity of DNA methyltransferases (Bönsch, et al., 2006; Miozzo et al., 2018).

Here, we elucidate the effects of moderate (20 mM) and severe (70 mM) subchronic ethanol (EtOH) exposure on early development by exposing hESCs to EtOH while differentiating them into the endodermal, mesodermal and ectodermal cells. Exposure time covers both pre- and post-implantation stages, i.e. from the differentiation of blastocyst-stage hESCs to differentiated germ layer cells in gastrulation. We explored the effects of EtOH on the transcriptome, methylome and metabolome of the embryonic germ layer cells.

## RESULTS

### Effects of EtOH on the transcriptome of germ layer cells

To study genome-wide EtOH-induced alterations in gene expression, we performed 3′ mRNA-sequencing (mRNA-seq) of three germ layers for control cells as well as for cells exposed to 20 mM and 70 mM EtOH (*n*=4 replicates/germ layer). When cells were exposed to 20 mM EtOH, differentially expressed genes (DEGs) were observed only in endodermal cells [79 DEGs of which 69 cells were down- and 10 cells upregulated; false discovery rate (FDR)<0.05] (Table S1).

#### Endodermal cells

**Fig. 1.**
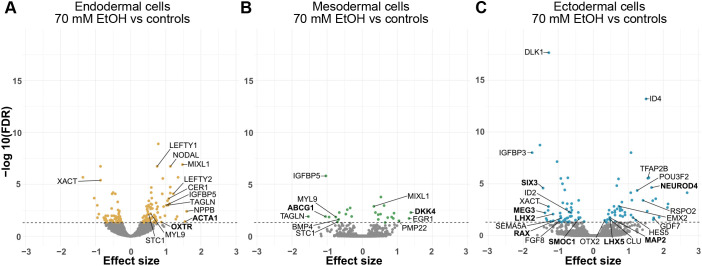
**Differential gene expression in 70 mM EtOH-exposed germ layer cells.** (A-C) Volcano plots showing the effect of 70 mM EtOH exposure on (A) mRNA expression in endodermal cells (control *n*=4, EtOH *n*=3), (B) mesodermal cells (control and EtOH *n*=3) and (C) ectodermal cells (control and EtOH *n*=4). Horizontal dashed lines indicate FDR<0.05. Common significantly altered genes between DNAm and mRNA-seq analyses in each germ layer are given in bold. Endodermal (orange), mesodermal (green) and ectodermal (blue) cells.

The largest number of significant changes in the germ layers was observed in 70 mM EtOH-exposed endodermal cells, including a total of 154 DEGs (67 down- and 87 upregulated). Of these, 30 DEGs were also found in cells exposed to 20 mM EtOH (Fig. 1A, Table S1). When 70 mM EtOH-exposed cells were compared to controls, highly upregulated expression of *nodal growth differentiation factor* (*NODAL*), *cerberus 1* (*CER1*) as well as *left-right determination factors 1* and *2* (*LEFTY1* and *LEFTY2*, respectively) were observed. They are all involved in the transforming growth factor beta (TGF-β) signaling pathway, as well as in left−right axis determination during the development of zebrafish embryo (Smith et al., 2011; Hashimoto et al., 2004). This process is crucial in establishing correct patterning for organ development and, interestingly, the loss of left−right symmetry as well as midline defects have been associated with PAE (Klingenberg et al., 2010). Also, we observed highly upregulated expression levels of *insulin like growth factor binding protein 5* (*IGFBP5*), which regulates growth and development of cells and tissues in the early mouse embryo (Salih et al., 2004).

**Fig. 2.**
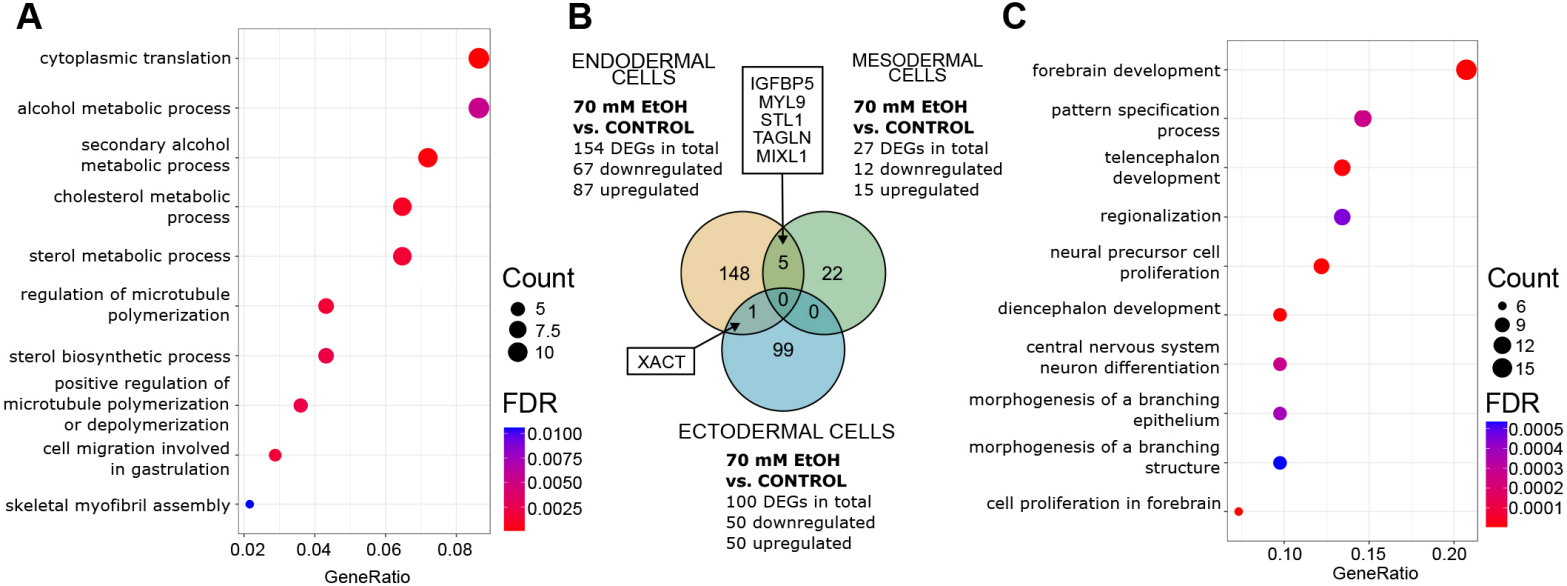
**Gene expression pathways and common genes of 70 mM EtOH-exposed germ layer cells.** (A) Significantly enriched terms identified in GO:BP enrichment analysis of 70 mM EtOH-induced DEGs in endodermal cells (FDR-corrected *P*-value<0.05). The ten most significant pathways are shown. (B) Venn diagram showing the number of 70 mM EtOH-induced DEGs that are common between germ layers. (C) Significantly enriched terms identified in GO:BP enrichment analysis of 70 mM EtOH-induced DEGs in ectodermal cells (FDR-corrected q-value<0.05). The ten most significant pathways are shown. Endodermal cells: control *n*=4, EtOH *n*=3; mesodermal cells: control and EtOH *n*=3; ectodermal cells: control and EtOH *n*=4.

To get an overall picture of the processes in which EtOH-induced DEGs cluster, we performed Gene Ontology (GO) pathway enrichment analysis for biological process (BP), molecular function (MF) and cellular component (CC). The most significant BP term for both concentrations of EtOH was the ribosome-mediated process ‘cytoplasmic translation’ (FDR-corrected *P*-value<0.05; Table S2). In the 70 mM EtOH-exposed endodermal cells, BP terms related to cholesterol metabolism, sterol metabolism, regulation of microtubule polymerization, cell migration involved in gastrulation and alcohol metabolism were also significant (Fig. 2A).

#### Mesodermal cells

The lowest number of significant changes was observed in mesodermal cells. We detected 27 DEGs (12 down- and 15 upregulated), including downregulated *BMP4*, *IGFBP5*, *TAGLN* and *ABCG1* as well as upregulated *DKK4* and *EGR1* in the 70 mM EtOH-exposed cells (Fig. 1B, Table S3). *Bone morphogenetic protein 4* (*BMP4*) serves as a key ligand for mesoderm formation (Tsaytler et al., 2023). Previously, reduced Bmp signaling has been observed in the heart cone and heart tube of EtOH-exposed zebrafish embryos (Sarmah et al., 2016). Furthermore, changes in gene expression and DNAm of *ATP-binding cassette sub-family G member 1* (*ABCG1*) in human and rat placenta (Auvinen et al., 2022; Zhang et al., 2019a) as well as changes in gene expression of *early growth response 1* (*EGR1*) in mouse embryonic forebrain have been linked to PAE (Subbana and Basavarajappa, 2022). Interestingly, despite the low number of DEGs in mesodermal cells, five of them, i.e. *IGFBP5*, *MIXL1*, *MYL9*, *STC1* and *TAGLN*, were in common with the endodermal cells (Fig. 2B). The upregulated transcription factor *mix paired-like homeobox* (*MIXL1*), is expressed in the primitive streak of the gastrulating embryo, playing a critical role in marking the cells to form mesoderm and endoderm (Ng et al., 2005).

#### Ectodermal cells

Based on effect sizes, the most prominent changes in gene expression were observed in the 70 mM EtOH-exposed ectodermal cells with 100 DEGs (50 down- and 50 upregulated) (Fig. 1C, Table S4). Among these DEGs were homeobox genes *EMX2*, *LHX2*, *LHX5*, *OTX2*, *POU3F2*, *RAX* and *SIX3*, growth-associated factors *GDF7*, *IGFBP3* and *FGF8*, as well as other developmentally essential genes, such as *DLK1*, *MEG3*, *SEMA5A*, *NEUROD4*, *ID2* and *ID4*. The transcription factor *orthodenticle homeobox 2* (*OTX2*), involved in formation of neuroectoderm during gastrulation (Rhinn et al., 1998; Larsen et al., 2010) and craniofacial development (Matsuo et al., 1995), has been previously associated with PAE in mouse brain (Kleiber et al., 2012). The transcription factor *six homeobox 3* (*SIX3*) is essential for forebrain formation and its expression − along with that of *EMX2*, *LHX2*, *LHX5* and *OTX2* − was altered by EtOH *in vitro* in a human pluripotent stem cell-based model of corticogenesis (Fisher et al., 2021). *Fibroblast growth factor 8* (*FGF8*) belongs to the FGF signaling pathway that is crucial for the development of various tissues and organ systems, including craniofacial, cardiovascular and brain structures (Xie et al., 2020) commonly affected in FASD (Hoyme et al., 2016). Moreover, previous PAE studies in human and mouse have reported alterations in the same genes as those identified in our current study, including *EMX2* (Rosenberg et al., 2010), *LHX2* (Subbanna and Basavarajappa, 2022), *ID2* (Abbott et al., 2018; Bottom et al., 2022; Conner et al., 2020; El Shawa et al., 2013; Hashimoto-Torii et al., 2011), *ID4* (Rosenberg et al., 2010; Boschen et al., 2021), *RSPO2* (Sambo et al., 2022), *MAP2* (Putzke et al., 1998) and *CLU* (Kim et al., 2012).

The most significantly downregulated gene, the paternally expressed *delta like non-canonical Notch ligand 1* (*DLK1*), is located within the *DLK1-DIO3* locus. Also, *maternally expressed 3* (*MEG3*) located within the same locus, was significantly downregulated. *DLK1* and *MEG*3 both belong to the group of imprinted genes, i.e. are genes that are epigenetically regulated according to the parent of origin in a locus-specific manner. This specific locus is necessary for fetal development and postnatal growth, and its non-coding RNAs (ncRNAs) are highly expressed in the early embryo (Mo et al., 2015; Prats-Puig et al., 2017). We identified one common DEG, i.e. *X active specific transcript* (*XACT*), expression of which was downregulated in 70 mM EtOH-exposed endodermal and ectodermal cells (Fig. 2B). *XACT* is a long ncRNA that plays a role in the control of X-chromosome inactivation (Vallot et al., 2013).

Ectodermal GO:BP pathways are closely related to the development of brain, nervous system and eyes. The most significant pathways include forebrain development, cell proliferation in forebrain, neural precursor cell proliferation, diencephalon development, telencephalon development, pattern specification and neuron differentiation of the central nervous system (FDR-corrected *P*-value<0.05; Table S5, Fig. 2C).

### Effects of EtOH on the methylome of germ layer cells

We used DNAm microarrays (Infinium MethylationEPIC BeadChip by Illumina) to explore the effects of 20 mM (*n=*3/germ layer) and 70 mM (*n*=4/germ layer) EtOH to the genome-wide DNAm of germ layer cells (control *n*=4/germ layer). We calculated genome-wide average DNAm (GWAM) by using all probes in the array and did not observe significant EtOH-induced alterations in total DNAm levels (Fig. S1). However, in 70 mM EtOH-exposed ectodermal cells, we observed significant hypomethylation at genomic location 1500 bp upstream of the transcription start site (TSS1500) (*P*=0.029, Wilcoxon rank-sum exact test; Fig. S1C). The global DNAm level was also predicted by comparing the mean DNAm levels of CpGs in repetitive elements (REs), such as Alu, endogenous retrovirus (ERV) and long interspersed nuclear element 1 (LINE1), that comprise 32% of the human genome in total (Zheng et al., 2017; International Human Genome Sequencing Consortium, 2001); and no changes in REs were observed between control and EtOH-exposed cells. Based on the analyses of EtOH-induced differentially methylated CpG sites, hereafter referred to as differentially methylated positions (DMPs) (with an FDR<0.05), we did not detect significant changes in the 20 mM EtOH-exposed germ layers. Therefore, we only report the results of the 70 mM EtOH exposure.

#### Endodermal cells

**Fig. 3.**
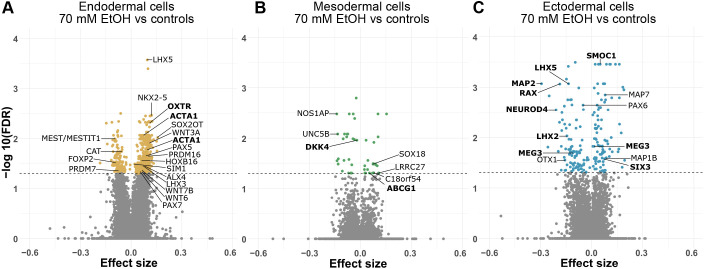
**Differential DNAm of 70 mM EtOH-exposed germ layer cells.** (A-C) Volcano plots showing the effect of 70 mM EtOH exposure on DNAm in (A) endodermal cells, (B) mesodermal cells and (C) ectodermal cells Horizontal dashed lines indicate FDR<0.05. Control and EtOH *n*=4/germ layer. Common significantly altered genes between DNAm and mRNA-seq analyses in each germ layer are given in bold. Endodermal (orange), mesodermal (green) and ectodermal (blue) cells.

Similar to our gene expression analysis results, the largest number of significant changes in DNAm analysis was observed in endodermal cells with 568 EtOH-induced DMPs (131 hypo- and 437 hypermethylated, associating with a total of 93 and 341 genes, respectively) (Fig. 3A, Table S6). Several homeobox genes (*ALX4*, *LHX3*, *LHX5*, *NKX2*-5, *HOXB13*, *PAX5* and *PAX7*) as well as some members of the WNT family (*WNT3A*, *WNT6* and *WNT7B*) − all of which play crucial roles for many developmental processes in gastrulation − were hypermethylated in endodermal cells. Also, genes encoding zinc finger proteins were found to be altered in endodermal cells, including hypomethylated *ZNF274*, *ZNF454*, *ZNF502*, *ZNF528*, *ZNF578* and *PRDM7*, as well as hypermethylated *PRDM16*. Their proteins function in transcriptional regulation during early embryonic development and cell differentiation (Cassandri et al., 2017). Both *PRDM7* and *PRDM16* encode histone methyltransferases and critical epigenetic regulators of early development (Pinheiro et al., 2012; Di Tullio et al., 2022). According to previous studies in mouse and zebrafish, *PRDM16* plays a main role in craniofacial development (Shull et al., 2020), abnormalities of which are characteristic of FASD (Hoyme et al., 2016). Furthermore, several altered genes − such as *ALX4* in the heart of (Abraham et al., 2022) and *SIM1* during early neurulation in (Liu et al., 2009) mouse embryos, as well as *FOXP2* and genes regulated via the *WNT* signaling pathway in human PAE placenta (Auvinen et al., 2022) − have previously been associated with EtOH exposure or PAE.

Moreover, 56 differentially methylated regions (DMRs) − defined as regions with a maximal allowed genomic distance of 1000 bp, containing three or more CpGs − were observed in endodermal cells (Table S7). The most significantly hypomethylated DMR as well as seven hypomethylated DMPs within regulatory and promoter regions were associated with imprinted paternally expressed *mesoderm specific transcript* (*MEST*) and its antisense RNA *MEST intronic transcript 1* (*MESTIT1*) sharing a promoter region with *MEST* (Nakabayashi et al., 2002). The most prominently hypermethylated DMP and two DMRs in the endodermal cells were in ncRNA *SOX2-OT*. The latter regulates expression of the developmentally critical transcription factor *SOX2*, which maintains pluripotency and directs differentiation into the germ layers (Masui et al., 2007; Zhang et al., 2019b). Interestingly, previous studies using mESCs (Ogony et al., 2013; Sánchez-Alvarez et al., 2013) and hESCs (Auvinen et al., 2022) suggest that EtOH can reprogram the lineage specification by changing the relative expression levels of pluripotency factors Oct4 and Sox2. Furthermore, we detected a hypomethylated DMR in the *CAT* gene and hypermethylated DMRs in *LHX5* and *NKX2-5*. Catalase, encoded by *CAT*, is an important antioxidant enzyme protecting against alcohol-induced damage and alleviating oxidative stress (Brocardo et al., 2011; Miller et al., 2013); it is also associated with PAE in mouse brain and heart (Drever et al., 2012; Atum et al., 2023).

**Fig. 4.**
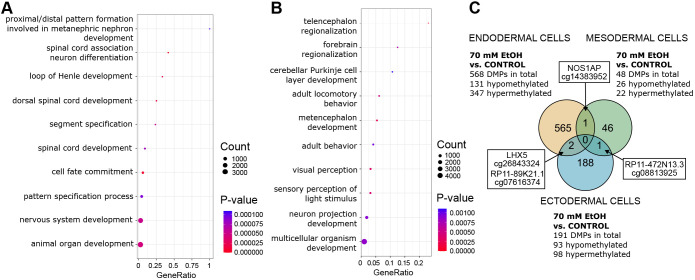
**DNAm pathways and common DMPs of 70 mM EtOH-exposed germ layer cells.** (A) Significantly enriched terms identified in GO:BP enrichment analysis of 70 mM EtOH-induced DMPs in endodermal cells (*P*<0.05). The ten most significant pathways are shown. (B) Significantly enriched terms identified in GO:BP enrichment analysis of 70 mM EtOH-induced DMPs in ectodermal cells (*P*<0.05). The ten most significant pathways are shown. (C) Venn diagram showing the number of 70 mM EtOH-induced DMPs that are in common between germ layers. Control and EtOH *n*=4/germ layer.

We performed GO pathway enrichment analysis for BP, MF and CC, and the most significant terms were related to MFs, such as transcription factor activity and transcription regulatory region binding (*P*<0.05; Table S8, Fig. 4A). Interestingly, there were also several significant terms related to neurodevelopment, such as spinal cord association neuron differentiation, nervous system development, generation of neurons and neuron differentiation.

#### Mesodermal cells

In line with the low number of alterations in mRNA-seq analysis, we observed only 48 EtOH-induced DMPs (26 hypo- and 22 hypermethylated associating with 14 and 15 genes, respectively) (Fig. 3B, Table S9) in the mesodermal cells. Additionally, six DMRs associating with *TOLLIP*, *ASB18*, *CHRND* and *DDR2* were observed (Table S10). The most prominently hypomethylated DMP was in *nitric oxide synthase 1 adaptor protein* (*NOS1AP*), which encodes a protein involved in neurotransmitter nitric-oxide synthesis (NOS) together with *NOS1*. Previous studies have reported a reduction of NOS activity in developing brain in PAE rodent models (Kimura et al., 1996; Kimura and Brien, 1998; Bonthius et al., 2002; de Licona et al., 2009). Moreover, *NOS1AP* was hypermethylated in the buccal epithelial cells of children with FASD (Portales-Casamar et al., 2016). The *NOS1AP* gene is also associated with several psychiatric disorders, such as attention-deficit/hyperactivity disorder (ADHD) (Freudenberg et al., 2015). Several other altered genes in the mesodermal cells are also associated with EtOH exposure in previous studies, such as *UNC5B*, *C18orf54* and *SOX18* in the gastrulation-stage mouse embryo (Boschen et al., 2021), *SOX18* in hESCs (Sánchez-Alvarez et al., 2013) as well as *LRRC27* and *DDR2* in human dental pulp stem cells (Hoang et al., 2016).

#### Ectodermal cells

We observed 191 EtOH-induced DMPs (93 hypo- and 98 hypermethylated) associating with 70 and 67 genes, respectively (Fig. 3C, Table S11), and 28 DMRs in the ectodermal cells (Table S12). Similar to DEGs in mRNA-seq analyses, there were fewer DMPs in the ectodermal cells compared to endodermal cells, but the ectodermal DMPs were more prominently altered based on their effect sizes. The most prominently hypomethylated DMP was in the 5′ untranslated region (5′ UTR) of *microtubule associated protein 2* (*MAP2*). This gene encodes a neuron-specific cytoskeletal protein that plays an important role in stabilizing dendritic shape, and is a known marker of dendrites and neuronal health (Dehmelt and Halpain, 2005; DeGiosio et al., 2022). *MAP2* has been associated with several neuropsychiatric and neurodegenerative disorders (DeGiosio et al., 2022), as well as alterations in EtOH-exposed rat brain and hippocampal cultured neurons (Putzke et al., 1998; Romero et al., 2010), and PAE rat frontal cortex (Tan et al., 1993). Additionally, two other microtubule-associated genes, *MAP1B* and *MAP7*, were both hypermethylated in the ectodermal cells.

Furthermore, several homeobox genes were altered by EtOH, including hypomethylated *LHX2*, *LHX5*, *PAX6*, *OTX1* and *RAX*, as well as hypermethylated *SIX3*. The transcription factor *LIM homeobox 2* (*LHX2*) regulates neural differentiation of hESCs via transcriptional modulation of *paired box 6* (*PAX6*) and *CER1* (Hou et al., 2013). *PAX6* has been associated with EtOH exposure or PAE in numerous studies (Mo et al., 2012; Fisher et al., 2021; Kim et al., 2010; Veazey et al., 2013; Aronne et al., 2008; Zhang et al., 2014; Peng et al., 2004; Yelin et al., 2007). *LHX2* and *PAX6* have previously been associated with PAE in the mouse forebrain (Subbanna and Basavarajappa, 2022), and *OTX1* has been associated with PAE in the mouse brain (Zheng et al., 2014).

As expected, GO pathway enrichment analysis of EtOH-induced DMPs revealed that almost all terms in ectodermal cells are related to brain and nervous system development, such as telencephalon regionalization, metencephalon development, visual perception and forebrain regionalization. Interestingly, we also saw BP terms related to adult locomotory behavior and adult behavior (Table S13, Fig. 4B).

Moreover, we compared EtOH-induced alterations between the germ layers and identified common DMPs. In endodermal and mesodermal cells, we found one common hypomethylated DMP, i.e. *NOS1AP* (cg14383952) (Fig. 4C). Also, *RP11-472N13.3* (cg08813925), a hypermethylated DMP associated with intergenic long ncRNA was observed in mesodermal and ectodermal cells. Furthermore, *LHX5* (cg26843324) and *RP11-89K21.1* (cg07616374), two common DMPs were identified in endodermal and ectodermal cells; both were hypermethylated in the endodermal but hypomethylated in the ectodermal cells.

Finally, gene expression results were compared with DNAm results. In the endodermal cells, two common genes were observed − *ACTA1* (five DMPs in TSS200 and TSS1500) and *OXTR* (DMP in 5′ UTR) − both of which were hypermethylated and upregulated. Mesodermal cells share two common genes between analyses, hypomethylated and upregulated *DKK4* (DMP in TSS1500), and hypermethylated and downregulated *ABCG1* (DMP in 5′ UTR). There were eight genes in common in the ectodermal cells, of which three − *LHX5* (two DMPs in gene bodies), *MAP2* (DMP in 5′ UTR) and *NEUROD4* (DMP in 5′ UTR) − were hypomethylated and upregulated, two hypermethylated and downregulated − *SIX3* (DMP in gene body) and *SMOC1* (DMP in gene body) − and two hypomethylated and downregulated − *LHX2* (three DMPs in TSS200, TSS1500 and 5′ UTR) and *RAX* (two DMPs in 3′ UTR)*.* In *MEG3*, two hypomethylated and one hypermethylated DMPs (DMPs in 5′ UTR) as well as downregulated gene expression were observed. We then compared these EtOH-induced gene expression and DNAm alterations to other early EtOH exposure models, including hESCs, mouse experiments and human cohorts (Table S14), as well as to studies of children with FASD diagnosis (Table S15).

### Effects of EtOH on the metabolomics of germ layer cells

To elucidate the effects of EtOH on the metabolome and the interplay between methylome, transcriptome and metabolome, we explored extracellular metabolites in the supernatants of the germ layer cells (endodermal and mesodermal supernatants *n=*4 replicates per condition and ectodermal supernatant *n=*5 replicates per condition). We used a non-targeted LC-MS metabolomics method (Klåvus et al., 2020) to measure a total of 9401 extracellular molecular features. The altered metabolic profiles were unique in each germ layer (Fig. S2), and a clear EtOH dose response between EtOH concentrations was observed. Similar trends of alterations were seen in the transcriptome, methylome and metabolome of the germ layer cells, with the largest quantitative changes occurring in the endodermal supernatant. Characteristics, statistical results, and reference spectra for all molecular features and identified metabolites are provided in the supplementary material (Table S16). Metabolite identification was focused on molecular features with *P<*0.05 in comparisons between control and EtOH-exposed samples. A total of 130 identified metabolites were classified into identification levels 1 and 2 according to Sumner et al. (2007) (Table S17).

#### Endodermal supernatants

**Fig. 5.**
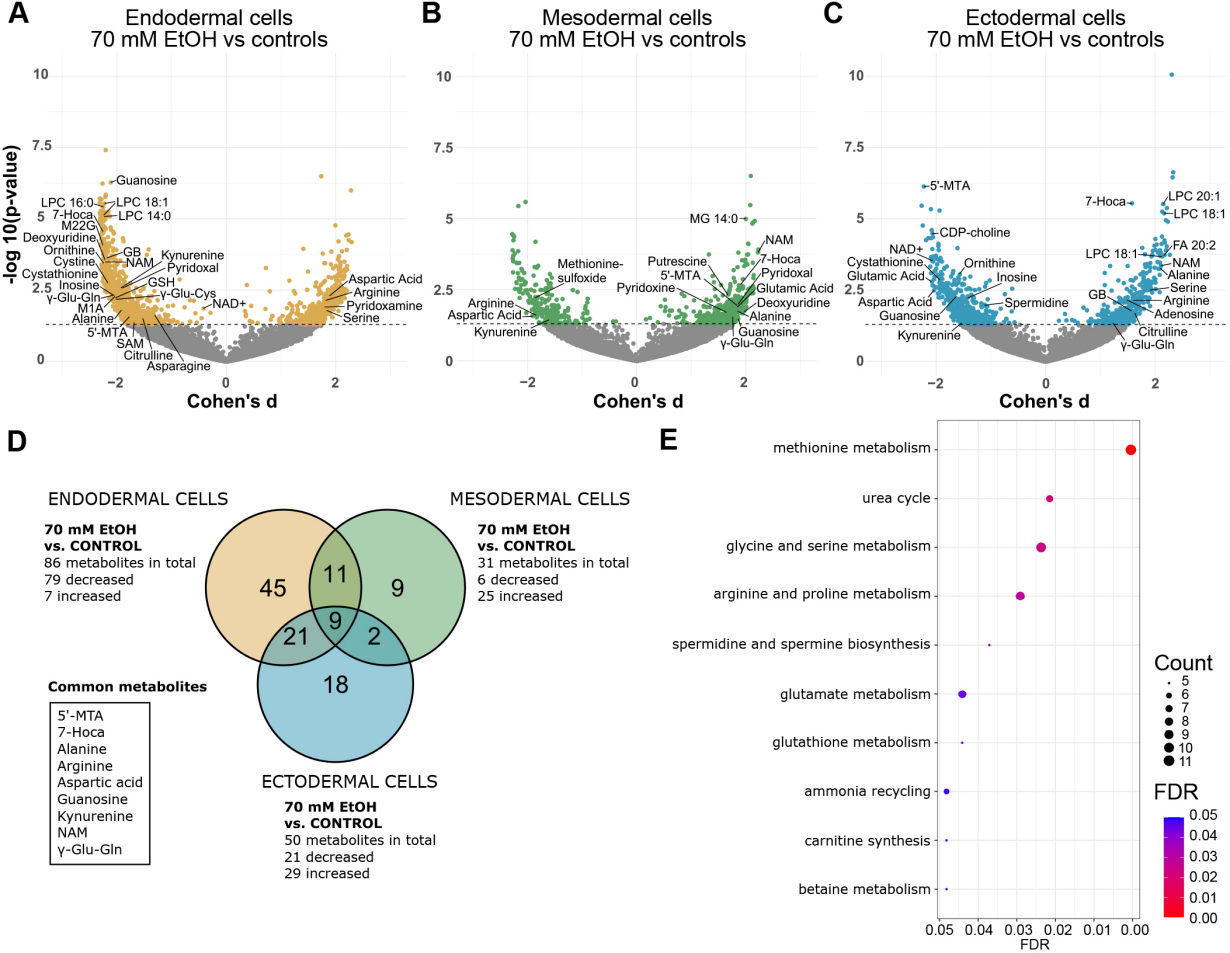
**Altered metabolites in 70 mM EtOH-exposed germ layers.** (A-C) Volcano plots showing the effect of 70 mM EtOH exposure on extracellular metabolites in (A) endodermal cells, (B) mesodermal cells and (C) ectodermal cells. Horizontal line marks *P*-value <0.05. (D) Venn diagram showing the numbers of 70 mM EtOH-induced alterations in annotated metabolites. (E) Significantly enriched terms identified in SMPDB metabolite set enrichment analyses of all EtOH-induced annotated metabolites (*P*<0.05). The ten most significant pathways are shown (FDR<0.05). 5′-MTA, 5′-methylthioadenosine; 7-Hoca, 7a-hydroxy-3-oxo-4-cholestenoic acid; GB, glycine betaine; GSH, reduced glutathione; LPC, lysophosphocholine; NAD+, nicotinamide adenine dinucleotide; NAM, N-acetylmethionine; M1A, 1-methyladenosine; M22G, N2,N2-dimethylguanosine; SAM, S-adenosylmethionine; γ-Glu-Cys, γ-glutamyl-cysteine; γ-Glu-Gln, γ-glutamyl-glutamine. Endodermal and mesodermal cells: control and EtOH *n*=4, ectodermal cells: control and EtOH *n*=5. Endodermal (orange), mesodermal (green) and ectodermal (blue) cells.

In the 20 mM EtOH-exposed endodermal cells, 62 metabolites with *P*<0.05 (Welch's *t*-test) and four metabolites with FDR<0.05 were observed, whereas, in cells of 70 mM exposure, we observed 86 metabolites with *P*<0.05 (Welch's *t*-test) and 32 metabolites with FDR<0.05 (Fig. 5A). Compared to mesodermal and ectodermal supernatants, the levels of altered metabolites in the endodermal supernatants were highly decreased, particularly in lysophosphocholines (LPCs) and lysophosphoethanolamines. Moreover, almost all identified amino acids, including the amino-acid derivative glycine betaine as well as nucleosides, were less abundant in endodermal cells, especially in cells of 70 mM EtOH exposure. Glycine betaine, synthesized from choline, is the principal methyl donor in the methionine cycle (Rosas-Rodríguez and Valunzuela-Soto, 2021).

Furthermore, the amounts of S-adenosylmethionine (SAM), a co-factor of DNA and histone methyltransferases, and nicotinamide adenine dinucleotide (NAD^+^), a co-factor of redox reactions, were significantly lower in the supernatants obtained from 70 mM EtOH-exposed endodermal cells compared to those of control cells (*P<*0.05). SAM is a principal methyl donor in various methylation reactions and cellular processes (Lu, 2000). Excessive alcohol consumption can decrease SAM levels, leading to aberrant DNAm patterns and alterations in gene expression (Varela-Rey et al., 2013). We also calculated the SAM:SAH ratio, commonly known as the methylation index, and observed a significantly decreased ratio in the supernatants obtained from 70 mM EtOH-exposed endodermal cells compared to those of control cells (*P*=0.036, Student's *t*-test; Fig. S3). The observed significant reduction in asparagine levels in the 70 mM EtOH-exposed endodermal cells are in line with previous studies, where heavy alcohol consumption reduced the amount of asparagine in human serum samples (Kärkkäinen et al., 2020). This decrease is also observed in the serum of mothers who consumed alcohol during the first trimester of pregnancy (Lehikoinen et al., 2018). Interestingly, the increase in acetaldehyde due to alcohol consumption may contribute to reduced asparagine levels in humans (John et al., 2024).

#### Mesodermal supernatants

In the 20 mM EtOH-exposed mesodermal cells, there were 20 metabolites with *P*<0.05 (Welch's *t*-test); in the 70 mM exposure, there were 31 metabolites with *P*<0.05 (Welch's *t*-test) and only one metabolite, monoacylglyceride MG 14:0, with FDR<0.05 (Fig. 5B). Notably, EtOH mainly increased metabolite levels in the 70 mM exposure, including two forms of vitamin B6 (pyridoxal and pyridoxine) and polyamine putrescine. Changes in activity of diamine oxidase (DAO), which is the rate-limiting enzyme in the terminal catabolism of polyamines, and the amounts of polyamines, such as putrescine and spermidine, have previously been associated with alcohol consumption and PAE in rats (Sessa and Perin, 1997).

#### Ectodermal supernatants

Similar to our gene expression findings, the observed alterations were evenly distributed between decreased and increased metabolite levels in the ectodermal cells. We observed 30 metabolites with *P*<0.05 (Welch's *t*-test) in the supernatants of 20 mM EtOH-exposed ectodermal cells, and 50 metabolites with *P*<0.05 (Welch's *t*-test) and eight metabolites with FDR<0.05 in the supernatants of 70 mM-exposed ones. Significantly decreased metabolites included 5′-methylthioadenosine (5′-MTA), cytidine diphosphate choline (CDP-choline) and NAD^+^, while significantly increased included three LPCs, the bile acid synthesis intermediate 7a-hydroxy-3-oxo-4-cholestenoic acid and fatty acid 20:2 (Fig. 5C).

N-acetylmethionine (NAM), a bioavailable source of methionine, was the only metabolite in this study, changed in all germ layers and both EtOH concentrations. NAM was significantly decreased in endodermal and significantly increased in mesodermal and ectodermal cell supernatants (*P<*0.05). For the 20 mM exposure, two altered metabolites were common among the germ layers: deoxyuridine and hydroxyphenyllactate. For the 70 mM exposure, eight common altered metabolites were identified: 7a-hydroxy-3-oxo-4-cholestenoic acid, 5′-MTA, alanine, aspartic acid, arginine, γ-glutamylglutamine, guanosine and kynurenine (Fig. 5D). 5′-MTA, the intermediate in the synthesis of adenine and methionine, produced by decarboxylation of SAM, was significantly less abundant in endodermal and ectodermal cells and more abundant in mesodermal cells compared to controls. Both 5′-MTA and kynurenine have neuroprotective effects in the human brain (Moreno et al., 2014; Vamos et al., 2009), and they have been associated with EtOH exposure in hESC-derived neural lineages (Palmer et al., 2012). Additionally, several studies have recently associated alterations in kynurenine with alcohol use disorder (Vidal et al., 2020; Leclercq et al., 2021; Jang et al., 2022; Mechtcheriakov et al., 2022).

We also observed several EtOH-induced alterations in the transsulfuration pathway, including changes in cystathionine, cystine, γ-glutamyl-cysteine, reduced glutathione (GSH) and oxidized glutathione. GSH is a known protector oxidative stress protector (Hayes and McLellan, 1999) and GSH levels have been found elevated in EtOH-exposed neonatal rat brain previously (Smith et al., 2005). Furthermore, spermidine levels and expression of spermidine acetyltransferase *SAT1* were both decreased. Spermidine has neuro- and cardioprotective properties (Madeo et al., 2018) and may mitigate the effects of PAE through its interaction with N-methyl-D-aspartate (NMDA) glutamate receptors (Honse et al., 2003; Savage et al., 1991). We also observed significantly decreased levels of the neurotransmitters glutamic acid and acetylaspartylglutamic acid in ectodermal cells, a finding in line with EtOH-induced disruptions of NMDA receptors. Also, a polymorphism in *SAT1* has previously been associated with alcohol use disorder (Vaquero-Lorenzo et al., 2014).

To predict metabolic pathways affected by EtOH, we performed metabolite set enrichment analyses (MSEA) by using the Small Molecule Pathway Database (SMPDB) library in MetaboAnalyst 5.0 (Pang et al., 2021) for all identified significantly altered metabolites together, and for each germ layer and EtOH concentration separately (Table S18). When analyzing all identified metabolites together, the most significant term was methionine metabolism (Fig. 5E). Methionine metabolism, urea cycle, glutathione metabolism, malate-aspartate shuttle, spermidine and spermine biosynthesis, and aspartate metabolism were significantly altered (*P*<0.05) in all 70 mM EtOH-exposed germ layers. Following the multiple testing correction (FDR<0.05), alterations were observed in the urea cycle, glycine and serine metabolism, and arginine and proline metabolism in 70 mM EtOH-exposed endodermal and ectodermal cells. Additionally, alterations in methionine metabolism, purine metabolism and malate-aspartate shuttle were observed in ectodermal cells, especially. However, this pathway analysis underestimates alterations in lipid metabolism, limiting the comprehensive understanding of alterations in the metabolome.

### Correlations between omics analyses

Finally, to understand the relationship between gene expression, DNAm and metabolites, relevant correlation analyses were conducted.

First, to elucidate the potential effects of DNAm on gene expression, we calculated correlations between 70 mM EtOH-exposed DEGs and the corresponding regulatory region (TSS1500, TSS200, 5′ UTR and 1stExon) probes for each gene separately. Our analysis revealed 111 significant correlations in the endodermal, 18 in the mesodermal and 78 in the ectodermal cells (*P*<0.05, Pearson correlation coefficient; Table S19a-c). Altogether 36% of the correlating regulatory region probes in the endodermal cells and 42% in the ectodermal cells were hypomethylated and correlated with upregulated genes. These included *LEFTY2* (two probes, *r=*−0.774 and −0.758; *P=*0.04 and 0.04, respectively), *MIXL1* (*r=*−0.9153; *P=*0.003) and *TAGLN* (*r=*−0.852; *P=*0.01) in the endodermal cells, as well as *NEUROD4* (three probes, *r=*−0.912, −0.882 and −0.774; *P=*0.001, 0.003 and 0.02, respectively), *RSPO* (*r=*−0.782; *P=*0.02), *NRXN3* (*r=*−0.732; *P=*0.003) and *MAP2* (*r=*−0.7844; *P=*0.02) in the ectodermal cells. Moreover, expression of *LHX2* and *MEG3* correlated with several probes within the regulatory regions in ectodermal cells. A total of eight correlated probes of downregulated *LHX2* were hypomethylated, and two correlated probes of downregulated *MEG3* were hypomethylated and six hypermethylated (Table S19c). Notably, downregulation of *DLK1*, locating in the same imprinted locus with *MEG3*, also correlated with one hypermethylated probe in the 5′ UTR (*r*=−0.838; *P*=0.009).

Second, to clarify the role of metabolism in DNAm and gene expression, we further tested whether the regulatory region probes that significantly correlated with DEGs also correlate with significantly altered metabolites. Significant correlations were observed only in the ectodermal cells: hypomethylation of *LHX2* correlated positively with CDP-choline and negatively with LPC 18:1 (18:1/0:0) (*r=*0.993 and −0.986*; P=*0.004 and 0.028 after Bonferroni correction, respectively; Pearson correlation coefficient), and hypermethylation of *SULF1* correlated negatively with guanosine (*r*=−0.933; *P*<0.004 after Bonferroni correction; Table S19d).

Third, we calculated correlations between 70 mM EtOH-induced DEGs and significantly altered metabolites (*P*<0.05). We found four significant correlations in endodermal, two in the mesodermal and nine in ectodermal cells (*P*<0.05 after Bonferroni correction, Pearson correlation coefficient; Table S19e-g). In ectodermal cells, the most significant correlation was observed between downregulated *spermidine/spermine n1-acetyltransferase 1* (*SAT1*) and the increased amount of LPC 18:1 (0:0/18:1) (*r*=−0.994; *P*<0.003). Furthermore, expression of downregulated *DLK1* correlated negatively with LPC 18:1 (0:0/18:1) and LPC 18:1 (18:1/0:0) (*r=*−0.991; *P=*0.008 and *P=*0.009, respectively), and the decreased expression of *SMOC1* correlated negatively with citric acid (*r*=−0.988; *P*=0.02).

Finally, we calculated correlations between 70 mM EtOH-induced DMPs and significantly altered (*P*<0.05) metabolites. We observed seven significant correlations in the endodermal cells, three in mesodermal and ten in ectodermal cells (*P*<0.05 after Bonferroni correction, Pearson correlation coefficient; Table S19h-j). In the endodermal cells, hypomethylation of *LHX5* correlated negatively with the level of ornithine (*r*=−0.997; *P*=0.04). In the ectodermal cells, hypomethylation of *LHX2* correlated positively with the level of CDP-choline (*r*=0.993; *P*=0.009), hypomethylation of *RAX* correlated negatively with the level of alanine (*r*=−0.991; *P*=0.02) and hypomethylation of *MEG3* correlated positively with LPC 20:1 (*r*=−0.988; *P*=0.04).

## DISCUSSION

### EtOH affects important developmental genes and signaling pathways

Here we utilized differentiating germ layer cells to study the effects of EtOH exposure on the transcriptome, methylome and metabolome during the developmentally crucial gastrulation stage. We observed significant alterations in several genes associated with the main morphogen signaling pathways during gastrulation and body patterning, such as WNT, FGF and BMP, the growth and differentiation factor (GDF) as well as TGF-β pathways. According to previous research, even small differences in signal duration or intensity during gastrulation can produce different responses, leading to switches in developmental trajectories (Gordon and Blobe, 2008). By affecting developmental programming, early PAE could disturb pattern formation and induce a wide spectrum of developmental abnormalities associated with the FASD phenotype. Indeed, it has been shown that a single dose of EtOH in gastrulation produced craniofacial malformations resembling the features of FASD in mouse (Lipinski et al., 2012; Parnell et al., 2009; Sulik, 2005).

The largest number of EtOH-induced changes in gene expression, DNAm and metabolites was observed in the endodermal cells. The majority of the common DEGs (26/30) were more prominently altered during severe EtOH exposure compared to moderate, reflecting a dose response. Among these DEGs was the developmentally essential cardiac hormone *natriuretic peptide B* (*NPPB*; also known as *BNP*) that was upregulated in 20 mM EtOH-exposed (effect size 1.2) and even more upregulated in 70 mM exposed cells (effect size 1.6). A higher amount of this known heart failure biomarker has been observed in the blood of heavy alcohol consumers compared to moderate consumers (Zile et al., 2016; Britton et al., 2020), which is in line with our results. Congenital heart diseases, including abnormal development of the heart and great vessels, are the most common birth defects worldwide (Zegkos et al., 2020). PAE during the first trimester affects heart development (Carmichael et al., 2003; Tikkanen and Heinonen, 1992), with an estimated 40% prevalence of congenital heart defects among individuals with FAS (Popova et al., 2016). Our findings support the early effects of EtOH on heart development, i.e. our observed EtOH-induced alterations in endodermal *NPPB*, *CAT* and *MAPK1* have also recently been detected in adult PAE mice hearts (Atum et al., 2023), and downregulation of mesodermal *BMP4*, a key inducer of early cardiac mesoderm (Tsaytler et al., 2023), has also been seen in EtOH-exposed zebrafish embryos (Sarmah et al., 2016). Coordination between endoderm and mesoderm is crucial for this first developing organ (Van Vliet et al., 2012; Ye et al., 2015) and, between them, five common DEGs − *IGFBP5*, *MIXL1*, *MYL9*, *STC1* and *TAGLN* − have all functions in the development of heart (Liu et al., 2012; Priya et al., 2020; Zawada et al., 2023).

### EtOH-induced aberrant FGF8 signaling in gastrulation

The most prominent EtOH-induced changes were observed in the ectodermal cells, which is consistent with the previously demonstrated sensitivity of the neurodevelopment to alcohol (Guerri, 2002; Popova et al., 2023). We observed EtOH-induced downregulation of ectodermal *FGF8* and *SIX3*, as well as upregulation of endodermal *LEFTY1* and *NODAL*, which follow the same altered gene expression patterns of zebrafish homologs when FGF signaling was interrupted. According to zebrafish studies, the lack of FGF signaling leads to the downregulation of *sixb3*, followed by upregulation of *lefty1*, which disrupts the normal development of central nervous system asymmetry (Neugebauer and Yost, 2014). Furthermore, we saw downregulation of ectodermal *DUSP6* and upregulation of endodermal *FGF17*. Both of these genes have essential roles in the FGF signaling pathway during early embryonic development (Tsukano et al., 2022; Li et al., 2007), and have been associated with EtOH exposure in mouse and hESCs (Sun et al., 2024; Khalid et al., 2014). In mice, mutations in *Fgf8* affect the formation of the corpus callosum (Stewart et al., 2016), which connects the left and right cerebral hemispheres and enables information transfer within the brain (Paul et al., 2007). Notably, based on magnetic resonance imaging studies, the corpus callosum in particular is commonly affected in individuals with FASD (Lebel et al., 2011). Our findings suggest an early origin for this developmental malformation and elucidate its potential molecular etiology.

Furthermore, *FGF8* and *SIX3* have been found to be mutated in holoprosencephaly (HPE) (Dubourg et al., 2016; McCabe et al., 2011; Wallis et al., 1999), a common developmental defect in midline patterning of the forebrain and/or midface (Dubourg et al., 2007; Marcorelles and Laquerriere, 2010). Approximately 1 in 250 embryos are affected by HPE, in which the complex etiology involves both genetic and environmental risk factors (Duborg et al., 2007; Matsunaga and Shiota, 1997). Indeed, based on previous studies, PAE is one of the environmental risk factors for HPE (Abe et al., 2018; Cohen and Shiota, 2002; Croen et al., 2000).

Additionally, we observed ectodermal EtOH-induced alterations in several genes, such as *LHX2*, *SHANK2*, *TRIO*, *ZMYND8*, *ARX*, *NRXN3* and *SEMA5A*, that have also been associated with autism spectrum disorder (ASD) (SFARI Gene Database, 2024; accessed: 1, August, 2024). This is in line with the observed overlapping phenotypes between FASD and ASD (Lange et al., 2018) and supports the existence of shared pathogenic mechanisms in these NDDs. Interestingly, haploinsufficiency of *LHX2* has been associated with non-specific NDD phenotype including ASD, variable intellectual disability, speech impairment and microcephaly, as well as behavioral, sleep and brain magnetic resonance imaging abnormalities (Schmid et al., 2023). Since all these features are also linked to the FASD phenotype, the role of *LHX2* in alcohol-induced NDD needs to be elucidated in future studies.

Moreover, we found several significant EtOH-induced alterations in the imprinted genes, which are essential for early development and growth as well as sensitive to environmental exposures (Weaver et al., 2009; Kappil et al., 2015), including PAE (Marjonen et al., 2018; Auvinen et al., 2022). We observed ectodermal correlations between hypermethylated probes, and downregulation of *DLK1* and *MEG3* in the imprinted *DLK1-DIO3* locus, which has previously been associated with both neurodevelopment (Mo et al., 2015; Isles, 2022) and PAE in mouse (Laufer et al., 2013). We also found DNAm changes in the imprinted genes in the endodermal cells: the most significantly hypomethylated DMR in *MEST* and hypermethylated DMP in *GNAS*.

### EtOH-induced alterations in metabolites associated with the methionine cycle

**Fig. 6.**
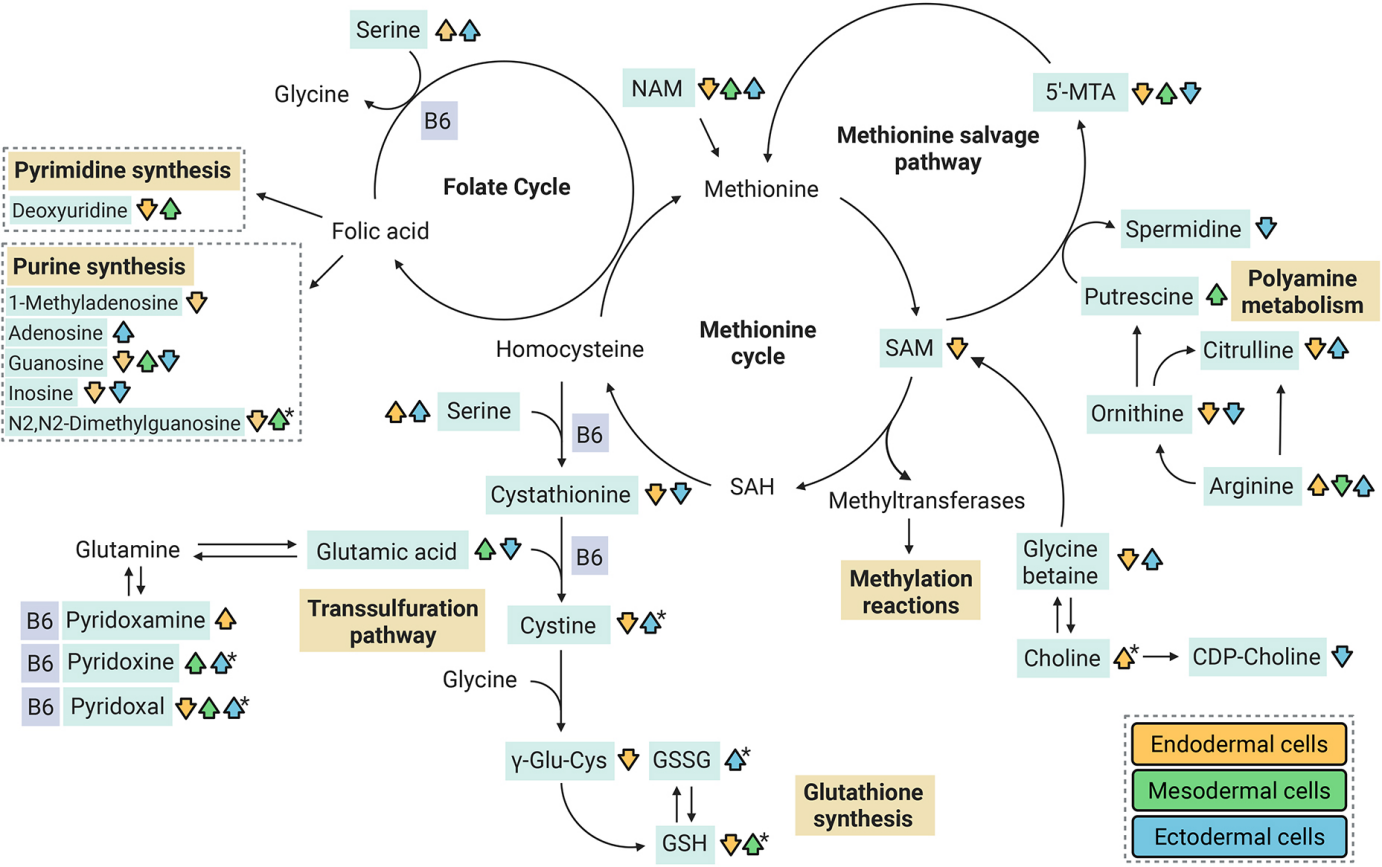
**Schematic of EtOH-induced alterations in metabolites of the methionine metabolism.** Arrowheads indicate significant EtOH-induced alterations in both exposures (20 and 70 mM EtOH) or in 70 mM EtOH exposure only. Arrowheads annotated with an asterisk indicate significant EtOH-induced alterations in 20 mM EtOH exposure only. Arrowhead colors indicate endodermal (orange), mesodermal (green) and ectodermal (blue) cells. Endodermal and mesodermal cells: control and EtOH *n*=4, ectodermal cells: control and EtOH *n*=5. 5′-MTA, 5′-methylthioadenosine; B6, vitamin B6; GSH, reduced glutathione; GSSG, oxidized glutathione; NAM, N-acetylmethionine; SAM, S-adenosylmethionine; γ-Glu-Cys, γ-glutamyl-cysteine. Created in BioRender by Wallén, E. (2025). https://BioRender.com/oqmd5jx. This figure was sublicensed under CC-BY 4.0 terms.

Consistent with previous results, several metabolites found to be significantly altered in this study have been associated with methionine metabolism, which provides methyl groups for the synthesis of DNA, polyamines, amino acids and phospholipids (Ducker and Rabinowitz, 2017). We observed various changes in the extracellular metabolites of the germ layers within different pathways of methionine metabolism, including the methionine, folate and methionine salvage cycles as well as the transsulfuration pathway (Fig. 6). Additionally, nucleosides, nucleotides and their derivatives, as well as several polyamines, amino acids and phospholipids were altered, indicating EtOH-induced disturbances in the methionine metabolism. In ectodermal and endodermal supernatants, we also observed reduced levels of NAD^+^ and, in endodermal cells, downregulation of *NMRK1* that encodes nicotinamide riboside kinase and acts as an NAD^+^ precursor. NAD^+^ has several important roles in methionine metabolism by serving as a co-factor for key enzymes, regulating methylation reactions, influencing polyamine synthesis and homocysteine recycling (Ducker and Rabinowitz, 2017; Covarrubias et al., 2021; Wang et al., 2022). Moreover, NAD^+^ acts as a coenzyme in EtOH metabolism (Zakhari, 2006).

Previously, it has been observed that, in liver, alcohol exposure inhibits the enzymatic activity within the methionine cycle (Kharbanda et al., 2007); also, decreased levels of folate have been reported in PAE placentas (Hutson et al., 2012). Indeed, mouse studies have demonstrated that methyl donors can play a protective role against PAE-induced epigenetic changes and adverse effects on the phenotype (Bekdash et al., 2013; Kwan et al., 2021), also in the early embryonic development (Breton-Larrivée et al., 2023). Similarly mitigating effects of choline supplementation on phenotype have been observed in human clinical trials (Jacobson et al., 2018; Wozniak et al., 2020; Warton et al., 2021). However, it is not known whether the additional supplements correct the lack of specific functionally essential methyl groups or whether they have a generally positive effect on development. Although the effects of EtOH on methyl donors in our study here varied between germ layers and despite the fact that culture medium contained excess amounts of methyl donors, we still detected alterations in choline, CDP-choline, glycine betaine and different forms of B6 vitamin.

### Effects of EtOH exposure on metabolism and gene regulation

Strong EtOH-associated correlations between gene regulation and metabolism indicate that the results of different omics analyses are closely interconnected in our *in vitro* model. Notably, correlations between genes and metabolites were especially robust in ectodermal cells. Also, the observed correlations were particularly strong in genes crucial for early development, including *DLK1*, *MEG3*, *LHX2*, *LEFTY2* and *NEUROD4*, together with the metabolite choline, which plays a vital role in the methionine cycle.

The impact of EtOH on the methionine cycle and, consequently, methylation potential is supported by the observed genome-wide hypomethylation within TSS1500 regulatory regions in the ectodermal cells. Also, in endodermal and ectodermal cells, a notable number of upregulated DEGs were correlated with hypomethylated probes in the regulatory regions, suggesting effects of EtOH on gene regulation. In contrast to our recent study using hESCs (Auvinen et al., 2022), we did not detect decreased expression of DNA methyltransferases *DNMT3A* or *DNMT3B* in this current study. Interestingly, although there were no EtOH-induced changes regarding methionine or SAH, a significant decrease in the SAM:SAH ratio was observed in endodermal cells. However, *in vivo* conditions are very different, and a disrupted methionine cycle combined with − often impaired − maternal nutrition (e.g. folate) can lead to substantially greater changes in the cellular methylation potential than those observed in this *in vitro* study.

In addition to DNAm, alcohol metabolism has been found to affect the regulation of fetal genes through histone acetylation in mice (Mews et al., 2019). This aligns with previous studies, finding that EtOH-induced alterations of gene expression involved in mouse cardiac development are primarily driven by increased histone acetylation rather than altered DNAm (Choudhury and Shukla, 2008; Kim and Shukla, 2006) − even under *in vitro* conditions (Zhong et al., 2010). Furthermore, in this current study, several metabolites and genes associated with the molecular mechanisms of acetylation (e.g. N-acetyllysine, *LNCPRESS1*, *CDK2AP1*, *ACAT2* and *SALL1*) were altered. This clearly indicates the presence of epigenetic mechanisms other than DNAm, and these other histone modifications, like histone acetylation, and small RNAs, should be considered in future studies.

### Limitations of the study and future prospects

This *in vitro* model enables examination of the effects of EtOH exposure on human cells under controlled conditions and excludes confounding factors in a challenging developmental stage of human research. We used the H1-hESC line, comprising well-characterized and extensively studied reference hESCs. However, by using only one male cell line, we cannot determine the effects of genetic background or sex on the observed EtOH-induced changes. Furthermore, in this relatively simple model, it is not possible to determine the causality or stability of the alterations, the impact of individual changes on developmental pathways, or obtain information about the effects of EtOH on the interactions of the germ layers and further development. Although we did not observe any signs of cell membrane damage potentially caused by *in vitro* exposure in phospholipids or visual differences in cell growth and structure between study groups, we cannot completely rule out method-related effects of EtOH. Unfortunately, we were only able to study extracellular metabolites from the same cells for which we conducted methylome and transcriptome studies, although EtOH-induced alterations in intracellular metabolomics would also be interesting. Many genes and metabolites altered by EtOH in the current study have been associated with PAE, FASD or EtOH exposure in human or other organisms, as described in several previous *in vivo* and *in vitro* studies, supporting our findings (Tables S14 and S15). The results that we presented here are associations and first steps towards understanding the effects of early PAE and the etiology of the variable FASD phenotype.

In addition to elucidating the etiology of alcohol-induced developmental disorders, our aim was to examine the sensitivity of gastrulation to alcohol exposure. Due to a large proportion (44–65%) of unplanned pregnancies worldwide (Bearak et al., 2018), there is a considerable risk of PAE prior to pregnancy recognition (Voutilainen et al., 2022). In this current study, both moderate (20 mM) and severe (70 mM) EtOH exposures induced changes in gene function and metabolism in a dose-dependent manner. Although statistically significant DNAm alterations were not observed in 20 mM EtOH-exposed cells, changes in metabolomes and endodermal gene expression highlight the sensitivity of early development to alcohol. The peak EtOH concentration of culture medium in moderate exposure corresponded to ∼0.9‰ and that of severe EtOH exposure to ∼3.2‰, which − together with previously observed 90% evaporation of EtOH during 24 h in cell culture dish (Rodriguez et al., 1992) − mimic human blood alcohol concentrations during subchronic binge drinking. However, the effects of *in vitro* and *in vivo* exposures cannot be directly compared. Considering the most prominent EtOH-induced changes in ectodermal cells in this as well as the sensitivity of neurodevelopment to EtOH observed in previous studies, the possibility of NDD caused by early PAE needs to be carefully investigated. Future studies employing advanced *in vitro* methods, such as embryo models and human forebrain organoids as well as appropriate human cohorts will clarify the effects of early PAE on human brain development and, consequently, on the phenotype.

## MATERIALS AND METHODS

### Maintenance and differentiation of hESCs and EtOH exposure

#### hESC culture

The human embryonic stem cell (hESC) line H1 (WA01) was obtained from Biomedicum Stem Cell Center (BSCC, Helsinki, Finland) through a license agreement with WiCell, Inc. hESCs were cultured in E8 Medium (Gibco) on Matrigel (Corning)-coated plates at 37°C and under 5% CO_2_, and tested for mycoplasma contamination. Culture medium was routinely replaced every day, and cells were passaged using 0.5 mM EDTA.

#### Germ layer cell differentiation

hESCs were differentiated into the three germ layers by using the STEMdiff Trilineage Differentiation kit (STEMCELL Technologies). Single-cell suspensions were prepared according to the manufacturer's instructions with 10 μM of the ROCK inhibitor Y-27632 (cat no.: 688000, Millipore) for the first 24 h after seeding, to promote the survival of cells. Cells were seeded in 6-well plates at the following densities: 2.5×10^5^ cells/well for mesodermal, 1×10^6^ cells/well for endodermal and 1.5×10^6^ cells/well for ectodermal cells. Differentiation was performed according to the manufacturer's instructions, and the STEMdiff Trilineage Differentiation media (cat no.: 05230, STEMCELL Technologies) were changed daily. Endodermal and mesodermal cells were collected after 5 days and ectodermal cells after 7 days.

#### EtOH exposure

The STEMdiff Trinileage Differentiation media were supplemented with EtOH (≥99.5 p-%) at a final concentration of 20 mM or 70 mM. Exposure was started at the same time as the differentiation (day 1). The exposure durations were 4 days (96 h) for endodermal and mesodermal cells, and 6 days (144 h) for ectodermal cells. The experiment was performed simultaneously with four or five replicates per germ layer. EtOH concentrations were selected based on previous publications (Nash et al., 2012; Khalid et al., 2014; Liyanage et al., 2015; De Filippis et al., 2016; Auvinen et al., 2022) and their biological relevance. The daily replacement of medium containing 20 mM or 70 mM EtOH, followed by a 90% loss of EtOH in 24 h by evaporation (Rodriquez et al., 1992) in unsealed culture dishes, mimics the pattern of subchronic moderate or severe binge drinking, corresponding to blood alcohol concentrations in humans of ∼0.9‰ or 3.2‰, respectively.

### DNA and RNA extractions

DNA and RNA were extracted simultaneously using AllPrep DNA/RNA/miRNA Universal Kit (Qiagen) according to the manufacturer's instructions. RNA quality was assessed using an Agilent 2100 Bioanalyzer (Agilent Technologies, Inc.), which was provided by the Biomedicum Functional Genomics Unit (FuGU) at the Helsinki Institute of Life Science and Biocenter Finland at the University of Helsinki, Finland.

### 3′ mRNA sequencing analysis

#### Differential expression analysis

Extracted RNA was prepared by diluting samples to 10 ng/μl and the library preparation for bulk 3′-sequencing of poly(A)-RNA was performed as previously described (Macosko et al., 2015) provided by FuGU. The libraries were sequenced on NextSeq 500 (Illumina) in three batches. Drop-seq pipeline was used to construct the 3′ mRNA sequencing (mRNA-seq) count table for RNA samples, resulting in the identification of 36,087 transcripts for downstream analysis. DESeq2 R package (Love et al., 2014) was used to detect DEGs (FDR-corrected *P*-value, *P*<0.05) separately for each germ layer with the model adjusting for batch. Principal component analysis revealed three outlier samples (one control and one 70 mM EtOH-exposed sample in mesodermal cells and one 70 mM EtOH-exposed sample in endodermal cell), which were removed from the analysis. Effect sizes for DEGs were defined as log2-fold changes calculated in DESeq2. The Benjamini−Hochberg procedure was used to control the FDR. Volcano plots and heat maps were plotted using ggplot2 and pheatmap R packages.

#### Pathway analysis

*enrichgo* function in R package clusterProfiler version 4.8.2. (Wu et al., 2021) was used to perform gene-set enrichment analysis for DEGs. The GO knowledgebase was used as the source for identifying significantly enriched BP, CC and MF terms, (FDR-corrected *P*-value<0.05). The Benjamini−Hochberg procedure was used to control the FDR. Due to the limited number of DEGs, pathway analysis was not performed for mesodermal cells.

### Validation of differentiation

To verify the identity of the established germ layers, we evaluated expression levels of key germ layer marker genes by using 3′ mRNA sequencing. Unexposed hESCs and germ layer cells exhibited characteristic expression levels corresponding to each cell type (Fig. S4), confirming successful trilineage differentiation. Further characterization with immunofluorescence analysis illustrated the expression of key germ layer markers in each germ layer (Fig. S5). The undifferentiated hESCs and differentiating stem cells were seeded onto 24-well culture plates with 30,000 cells per well prior to immunostaining. Cells were fixed in 4% paraformaldehyde (Fisher Chemical) in PBS for 10 min, then washed with PBS. The cells were permeabilized by 0.5% Triton X-100 in PBS for 10 min and treated with Ultra Vision (UV)-blocker (Thermo Scientific) for 10 min. Immunostaining was performed using the following antibodies: anti-SOX17 (R&D systems, cat. no.: AF1924, goat, polyclonal dilution 1:500) for endodermal cells, anti-NCAM (EMD Millipore, cat. no.: AB5032, rabbit, monoclonal dilution 1:500) for mesodermal cells, anti-PAX6 (Thermo Fisher Scientific, cat. no.: 42-6600, rabbit, polyclonal dilution 1:400) for ectodermal cells. Primary antibodies were diluted in 0.1% Tween in PBS, added to the wells and incubated for 24 h in the dark at 4 °C on a Stuart SSL4 see-saw rocker. Secondary antibodies [PAX6 and NCAM: anti-rabbit IgG (H+L) Alexa Fluor 488 (Thermo Fisher Scientific, cat. no: A21206, host species donkey, dilution 1:500), SOX17: anti-goat IgG (H+L) Alexa Fluor 488 (Thermo Fisher Scientific, cat. no.: A11055, host species donkey, dilution 1:500)] were diluted in 0.1% Tween in PBS and added to the wells. Cells were imaged using the EVOS FL Cell Imaging System (Thermo Fisher Scientific).

To examine the potential heterogeneity of control and EtOH-exposed germ layer cell populations, we investigated the expression of several cell population-specific marker genes (endodermal cells: R&D Systems, https://www.rndsystems.com/research-area/early-endodermal-lineage-markers, accessed 30 April 2025, mesodermal cells: Zhao et al., 2025, ectodermal cells: R&D Systems https://www.rndsystems.com/research-area/ectodermal-lineage-markers and https://www.rndsystems.com/research-area/neural-progenitor-cell-markers, accessed 30 April 2025). Based on expressions, we did not detect signs of specific cell populations (endoderm: primitive or definitive; mesoderm: early, axial or advanced; ectoderm: early ectoderm, neural progenitor cells or ectodermal surface) (Fig. S6).

### DNAm microarrays

#### Genome-wide DNAm analysis

In control and 70 mM EtOH-exposed germ layers, genomic DNA (1000 ng) was sodium bisulfite-converted using the Zymo EZ DNAm™ kit (Zymo Research, Irvine, CA, USA). Genome-wide DNAm was assessed with Infinium Methylation EPIC BeadChip v1.0 (Illumina, San Diego, CA, USA), following standard the protocol at the Institute for Molecular Medicine Finland, Helsinki, Finland. The raw DNAm dataset was pre-processed, quality-controlled and filtered with ChAMP R package (Tian et al., 2017) using the minfi method (Fortin et al., 2017). Data were filtered using detection *P*-values and bead count values with default thresholds. Probes were normalized with noob (Triche et al., 2013) in minfi R package (Fortin et al., 2017), and BMIQ in wateRmelon R package (Pidsley et al., 2013). Following normalization, probes located in sex chromosomes and probes binding to polymorphic and off-target sites (McCartney et al., 2016; Pidsley et al., 2016) were filtered. After filtering steps, 756 351 probes were retained for further downstream analysis. Annotation information was merged to corresponding probes from IlluminaHumanMethylationEPICanno.ilm10b4.hg19 R package, which is based on the file ‘MethylationEPIC_v-1-0_B4.csv’ from Illumina. The following abbreviations were used: TSS1500, TSS200, 5′ UTR, 3′ UTR, N_shelf: north shelf, N_shore, S_shore, S_shelf: south shelf. Probes were annotated to genes based on UCSC and GENCODE Comprehensive V12 (GencodeComp) databases. Gene names were used primarily according to UCSC, and the locations of probes were primarily determined according to GencodeComp. In the control and 20 mM EtOH-exposed germ layers, DNAm analysis performed with the following changes: Genomic DNA (250 ng) was sodium bisulfite-converted and genome-wide DNAm was assessed with Infinium Methylation EPIC BeadChip v2.0 (Illumina, San Diego, CA, USA). The raw DNAm dataset was preprocessed and quality-controlled with the minfi R package (Fortin et al., 2017). Following normalization, probes located within sex chromosomes and cross-reactive probes were filtered. Cross-reactive probes were identified from the expanded manifest file as described by Peters et al. as probes with ‘Y’ in the ‘CH_WGBS_evidence’ field Peters et al. (2024). After filtering steps, 860,510 probes were retained for further downstream analysis. Annotation information was merged to corresponding probes from IlluminaHumanMethylationEPICv2anno.20a1.hg38 R package, which is based on the file ‘EPIC-8v2-0_A1’ from Illumina. The following abbreviations were used: TSS1500, TSS200, 5′ UTR, 3′ UTR.

#### GWAM analysis

β-values of all normalized probes in the array were used to calculate sample-wise GWAM levels (Li et al., 2018), similarly for both EPICv1 and EPICv2 samples. Differences between EtOH-exposed and control samples were calculated by selecting the appropriate statistical test from two-tailed Student's *t*-test, Welch two sample *t*-test or Wilcoxon rank-sum exact test based on normality and variances. Normality of the data was assessed with Shapiro−Wilk normality test and the variances were compared with F-test. Gene locations were used according to UCSC, and any probe with missing location information was marked as ‘unknown’. In case of multiple location entries, group ‘others’ was used. For EPICv2, probes annotated to exons other than the first exon were marked as ‘body’.

#### RE DNAm analysis

Processed DNAm data (M-values) was used to predict DNAm in Alu, ERV and LINE1 elements sample-wise using a Random Forest-based algorithm implemented by REMP R package (Zheng et al., 2017). For EPICv2, only the corresponding probes available in EPICv1 data were included in the analysis. Additionally, duplicate probes in EPICv2 were removed with aggregate_to_probes function from IlluminaHumanMethylationEPICv2anno.20a1.hg38 R package, which retains one probe with a mean methylation value from each set of duplicate probes. EPICv1 data were analyzed with hg19 annotation and EPICv2 data with hg38 annotation but, otherwise, the analysis was performed similarly. Less reliable predicted results were trimmed according to the default quality score threshold 1.7 and missing rate 0.2 (20%). Differences between EtOH-exposed and control samples were calculated by selecting the appropriate statistical test from two-tailed Student's *t*-test, Welch’s two-samples *t*-test or Wilcoxon rank-sum exact test based on normality and variances. Normality of the data was assessed with Shapiro−Wilk normality test and the variances were compared with *F*-test.

#### DMP analysis

DMP analysis was performed by Limma R package (Ritchie et al., 2015) by using M-values. The design matrix included no covariates for EPICv1 data, but for EPICv2 data plate was used as a covariate. β-values were used for visualization and interpretation of the results. Effect sizes for DMPs were defined as β-value log2 fold changes calculated in Limma. DMPs were considered significant when the FDR-corrected *P*-value was <0.05. Benjamini−Hochberg procedure was used to control FDR. Volcano plots were plotted using ggplot2 R package.

#### DMR analysis

DMRcate R package was used for analysing DMRs (Kolde et al., 2016) in the EPICv1 data. In short, analysis in DMRcate began with standard linear modeling, producing t-values for each CpG site. DMRcate then applied kernel smoothing, which factored in the neighboring sites, giving weighted average values for each CpG site. A model for the smoothed data was then created and the CpG sites with adjusted *P*-values below the threshold were agglomerated into regions. DMRcate was set to determine probes (≥3) in a region with a maximal allowed genomic distance of 1000 bp, and scaling factor C set at 2, as per authors' recommendation (Kolde et al., 2016). Default *P*-value cut-offs, including FDR<0.05 for individually significant CpGs, were used in the analysis.

#### Pathway analyses

Enrichment analysis was performed for significant DMPs (*P*<0.05) by *gometh* function in missMethyl R package (Phipson et al., 2015), which considers the different number of probes per gene present on the EPIC array and CpGs that are annotated to multiple genes. missMethyl was set to use the GO database as the source for identifying significantly enriched BP, CC, and MF terms. Terms with a nominal *P*<0.05 were reported because the terms were significant with FDR correction only in the endodermal cells. Due to the low number of DMPs in the 70 mM EtOH-exposed mesodermal cells and DMRs in all germ layer cells, as well as in all 20 mM germ layer cells, they were not included in pathway analyses.

### Metabolomic analysis

#### Sample processing

Metabolomics analysis was conducted on the supernatants of the same samples used in the DNAm and gene expression analysis. For metabolite extraction, samples were randomized and 400 µl of cold methanol (100%) was added to 100 µl of sample. Samples were kept on ice between the steps. The pooled quality control (QC) sample was prepared by combining 10 µl from each sample. Supernatants were filtered (Acrodisc 4 mm with 0.45 μm membrane) and inserted into HPLC vials for analysis.

#### LC–MS metabolite profiling analysis

Samples were analyzed by liquid chromatography–mass spectrometry (LC-MS), consisting of an ultra-high performance liquid chromatography (TUPLC) combined with Thermo Q Exactive™ Hybrid Quadrupole-Orbitrap mass spectrometer (Thermo Scientific). Technical specifications of the reverse phase (RP) column were: Zorbax Eclipse XDB-C18, particle size 1.8 µm, 2.1×100 mm (Agilent Technologies). For the hydrophilic interaction chromatography (HILIC) column, technical specifications were: Acquity UPLC BEH Amide 1.7 µm, 2.1×100 mm (Waters Corporation). The chromatography parameters were as follows: for RP chromatography, the column oven temperature was set to 50°C, and the flow rate to 0.4 ml/min gradient elution with UltraPure H_2_O (eluent A) and methanol (eluent B) both containing 0.1% (v/v) of formic acid. Gradient profile for RP separations was 0 to 10 min: 2% B→100% B; 10−14.5 min: 100% B; 14.5−14.51 min: 100% B→2% B; 14.51−16.5 min: 2% B. For HILIC, the column oven temperature was set to 45°C, flow rate 0.6 ml/min, gradient elution with 50% v/v acetonitrile (ACN) in UltraPure H_2_O (eluent A) and 90% v/v ACN in UltraPure H_2_O (eluent B), both containing 20 mM ammonium formate (pH 3). The gradient profile for HILIC separations was 0 to 2.5 min: 100% B, 2.5−10 min: 100% B→0% B; 10−10.01 min: 0% B→100% B; 10.01−12.5 min: 100% B. Both negative and positive electrospray ionization (ESI) were used for both analytical modes. ESI settings were ray voltage 3.5 kV for positive and 3.0 kV for negative, max spray current 100, flow rates 40 for sheath gas, 10 for auxiliary gas and 2 for spare gas (as arbitrary units for ion source), S-lens RF level 50 V, capillary and probe heater temperature 300°C. A full scan range from m/z 60 to 700 for HILIC modes and 120 to 1200 for RP modes with a resolution of 70,000 (m/Δm, full width at half maximum at 200 u) and an automated injection time and gain control targeted at 1,000,000 ions. For tandem mass spectrometry (MS/MS), three peaks with apex trigger 0.2−3 s were selected for MS/MS fragmentation with 15 s dynamic exclusion. Normalized collision energy at 20, 30 and 40%, was used in MS/MS with a mass resolution of 17,500 (m/Δm, full width at half maximum at 200 u), an automated gain targeted at 50,000, and an isolation window 1.5 m/z.

#### Data analysis

Peak detection and alignment were performed in MS-DIAL ver 4.90 (Tsugawa et al., 2020). For the peak collection, full scale of m/z values for each mode were considered, and retention times after 0.5 min were considered. The amplitude of the minimum peak height was set at 300,000 in the negative modes or 500,000 in the positive modes. The peaks were detected using the linear weighted moving average algorithm. For the alignment of the peaks across samples, the retention time tolerance was 0.1 min, and the m/z tolerance was 0.01 Da. Drift correction and removal of low-quality signals were done with Notame R-package as described in Klåvus et al. (2020). For feature-wise analysis, we used Welch's *t*-test and Cohen's d-effect sizes.

#### Compound identification

The chromatographic and mass spectrometric characteristics (retention time, exact mass and MS/MS spectra) of the significantly differential molecular features were compared with entries in an in-house standard library and publicly available databases, such as METLIN and The Human Metabolome Database (HMDB), as well as with published literature. The annotation of each metabolite and the level of identification was given based on the recommendations published by the Chemical Analysis Working Group (CAWG) Metabolomics Standards Initiative (MSI) (Sumner et al., 2007).

#### Pathway analyses

MetaboAnalyst 5.0 was used for the metabolite set enrichment analyses (MSEA) with The Small Molecule Pathway Database (SMPDB) library of the annotated metabolites (Pang et al., 2021). Enrichment analysis was performed on the metabolites significantly altered by EtOH (*P*<0.05) with an available HMDB ID as follows: 40 metabolites in 20 mM EtOH-exposed endodermal cells, 33 metabolites in 70 mM endodermal cells, 15 metabolites in 20 mM mesodermal cells, 27 metabolites in 70 mM mesodermal cells, 21 metabolites in 20 mM ectodermal cells, 55 metabolites in 70 mM ectodermal cells and 79 metabolites in all germ layer cells together.

### Correlation analysis

Correlations between DEGs and CpGs in the corresponding regulatory region for each gene, DEGs and differentially altered annotated metabolites, as well as DMPs and differentially altered annotated metabolites were calculated with Pearson correlation. β-values of CpGs, variance stabilizing transformed (vst) expression counts, and signal intensities of molecular features were used. In the analysis, CpGs annotated for TSS1500, TSS200, 5′ UTR and 1stExon regions, based on GencodeComp annotation, were considered as regulatory region CpGs. Analysis for correlation between DEGs and the corresponding regulatory region CpGs was calculated for each gene individually, and thus unadjusted *P*-values were used to determine the significance of results. Bonferroni correction was used for multiple testing correction in all other analyses due to the large number of comparisons.

## Supplementary Material

10.1242/dmm.052150_sup1 Supplementary information

Table S1. EtOH-induced DEGs in the endodermal cells analyzed by mRNA-seq

Table S2. GO pathway analysis of DEGs in the endodermal cells

Table S3. EtOH-induced DEGs in the mesodermal cells analyzed by mRNA-seq

Table S4. EtOH-induced DEGs in the ectodermal cells analyzed by mRNA-seq

Table S5. GO pathway analysis of DEGs in the ectodermal cell

Table S6. EtOH-induced DMPs in the endodermal cells analyzed by microarrays

Table S7. EtOH-induced DMRs in the endodermal cells analyzed by microarrays

Table S8. GO pathway analysis of endodermal DMPs

Table S9. EtOH-induced DMPs in the mesodermal cells analyzed by microarrays

Table S10. EtOH-induced DMRs in the mesodermal cells analyzed by microarrays

Table S11. EtOH-induced DMPs in the ectodermal cells analyzed by microarrays

Table S12. EtOH-induced DMRs in the ectodermal cells analyzed by microarrays

Table S13. GO pathway analysis of ectodermal DMPs

Table S14. Common genes in the current study and previous early alcohol exposure gene expression and/or DNAm studies

Table S15. Common genes in the current study and previous DNAm studies of children with FASD

Table S16. Characteristics, statistical results, and reference spectra for all metabolic features

Table S17. EtOH-induced significantly altered extracellular metabolites of all germ layer cells.

Table S18. SMPDB enrichment analysis of the annotated metabolites significantly altered by EtOH.

Table S19. Correlations between omics analyses
