# Supplementary material for: Effects of alcohol on the transcriptome, methylome and metabolome of in vitro gastrulating human embryonic cells
Source: Dis Model Mech. 2025 Jun 18;18(6):dmm052150. doi: 10.1242/dmm.052150 (PMC12208196; doi:10.1242/dmm.052150)
Supplement: Supplementary information [file dmm-18-052150-s1.pdf]

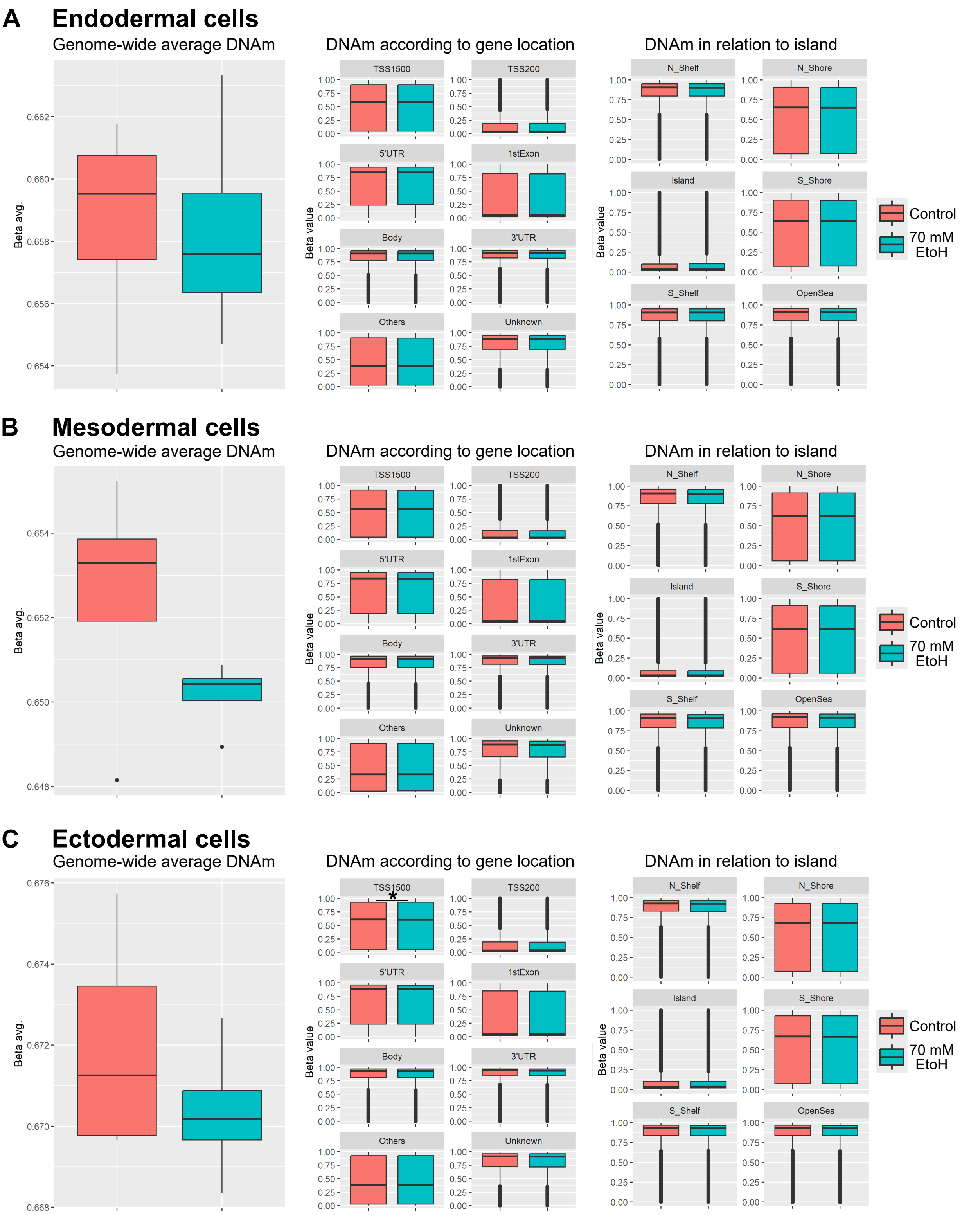

Fig. S1. GWAM comparison between controls and 70 mM EtOH-exposed germ layer cells. Comparison of DNAm in all probes, in relation to gene, and in relation CpG island in **a** endodermal cells, **b** mesodermal cells, and **c** ectodermal cells. *$P < 0.05$, Student's t test or Welch two sample t test. Control and EtOH n = 4/germ layer. TSS1500: 1500 bp upstream of transcription start site, TSS200: 200 bp upstream of TSS, UTR: untranslated region, N_shelf: north shelf, N_shore: north shore, S_shore: south shore, S_shelf: south shelf.

Disease Models & Mechanisms • Supplementary information

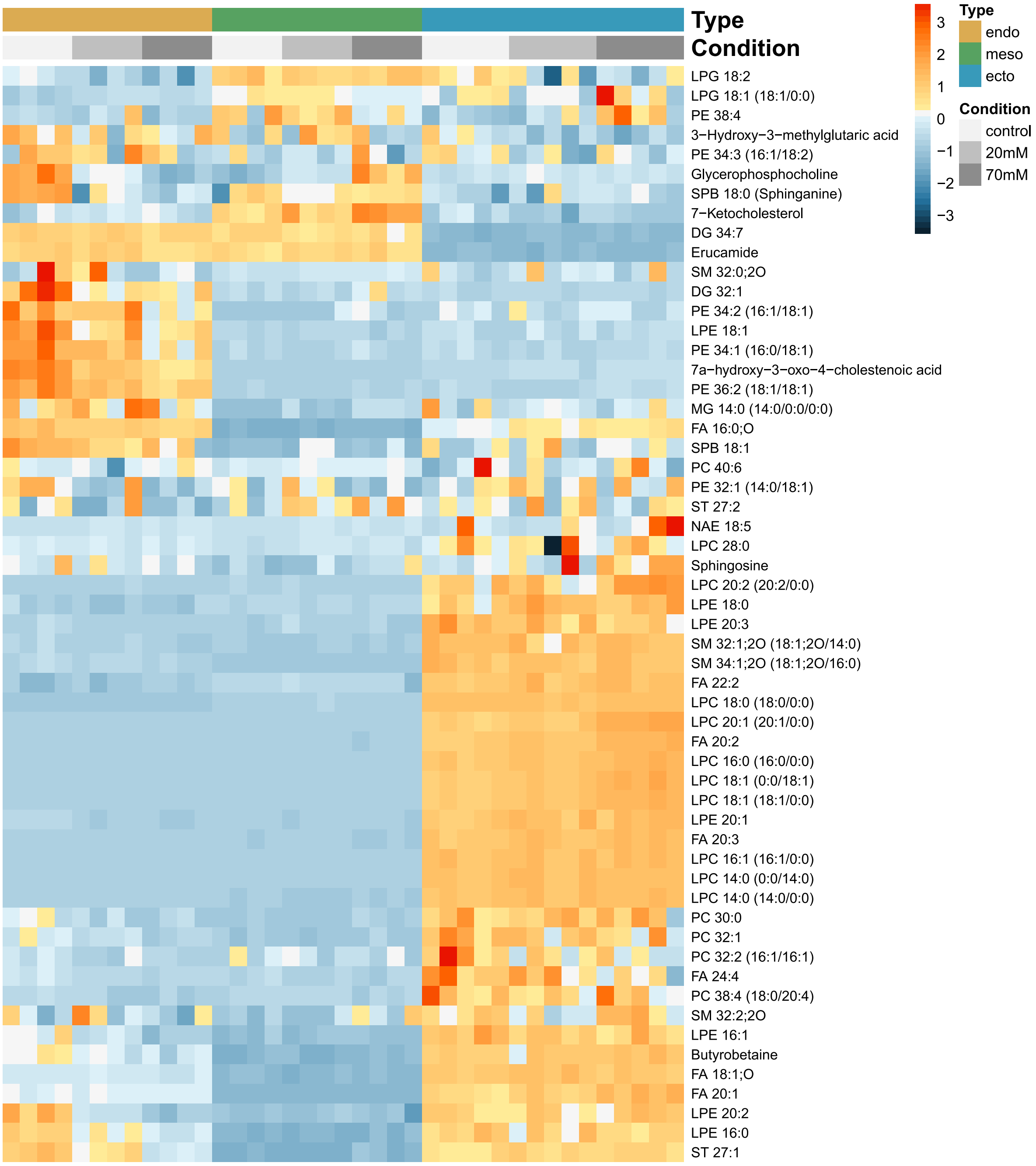

Disease Models & Mechanisms • Supplementary information

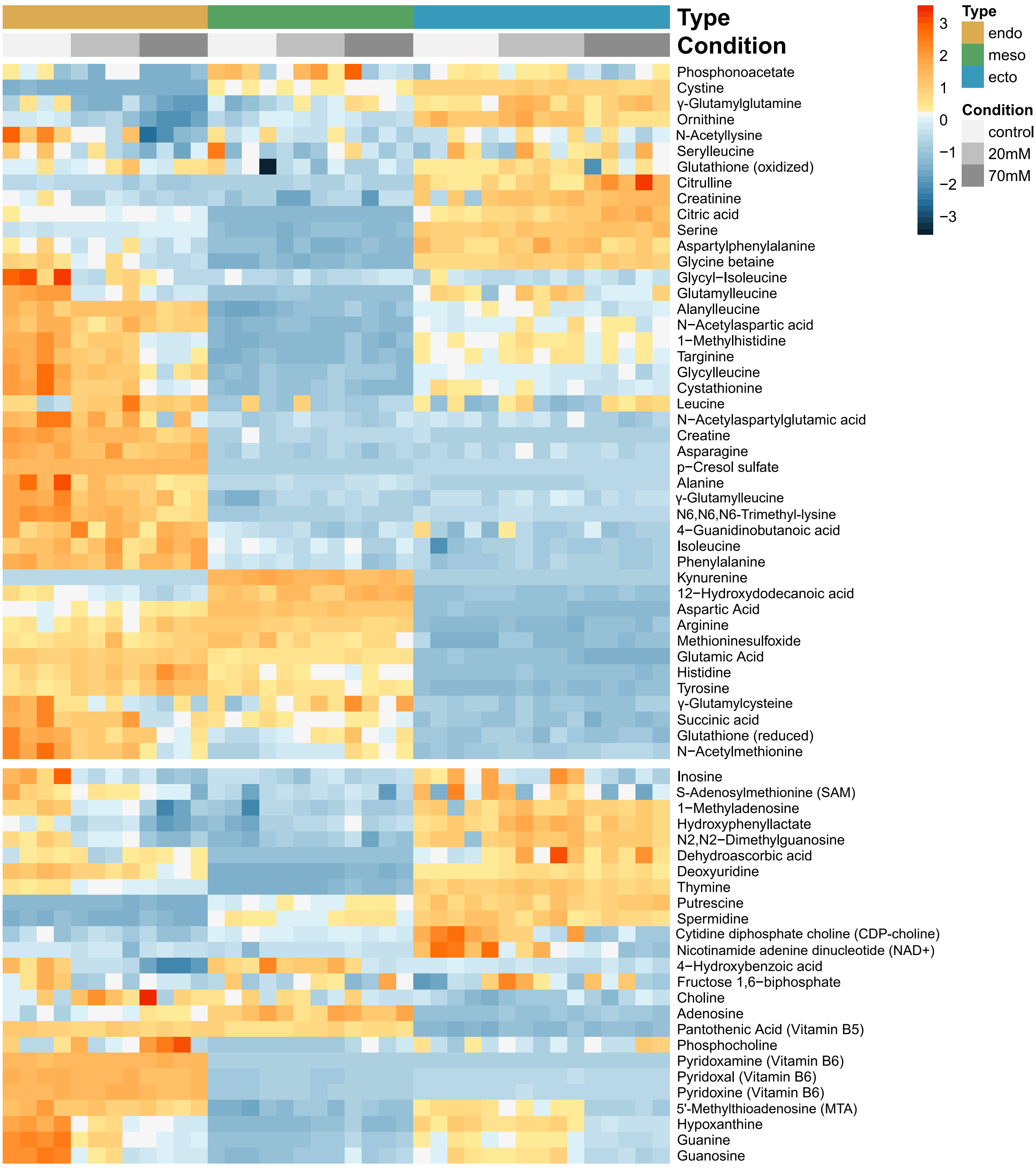

Fig. S2. Heat map of the normalized abundances of all annotated metabolites in the germ layers. Hierarchical clustering was applied to arrange the metabolites based on their similarity of the abundance between the samples. Endodermal and mesodermal cells *n* = 4/condition/germ layer, ectodermal cells *n* = 5/condition/germ layer.

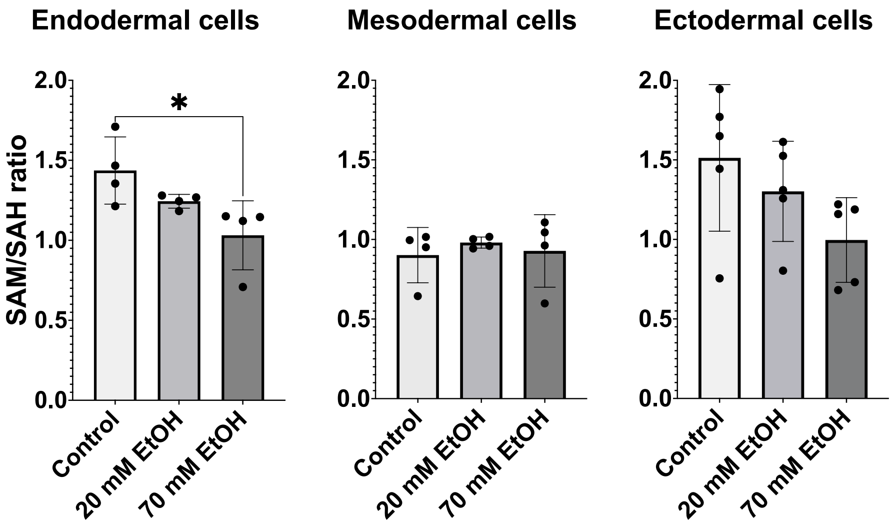

Fig. S3. Ratio of SAM/SAH in the germ layers.

Ratio of S-adenosylmethione (SAM) and S-adenosylhomocysteine (SAH). Ratio presented as mean ±SD. Endodermal and mesodermal cells *n* = 4/condition/germ layer, ectodermal cells *n* = 5/condition/germ layer. *P-value < 0.05, **P-value < 0.01.

Disease Models & Mechanisms • Supplementary information

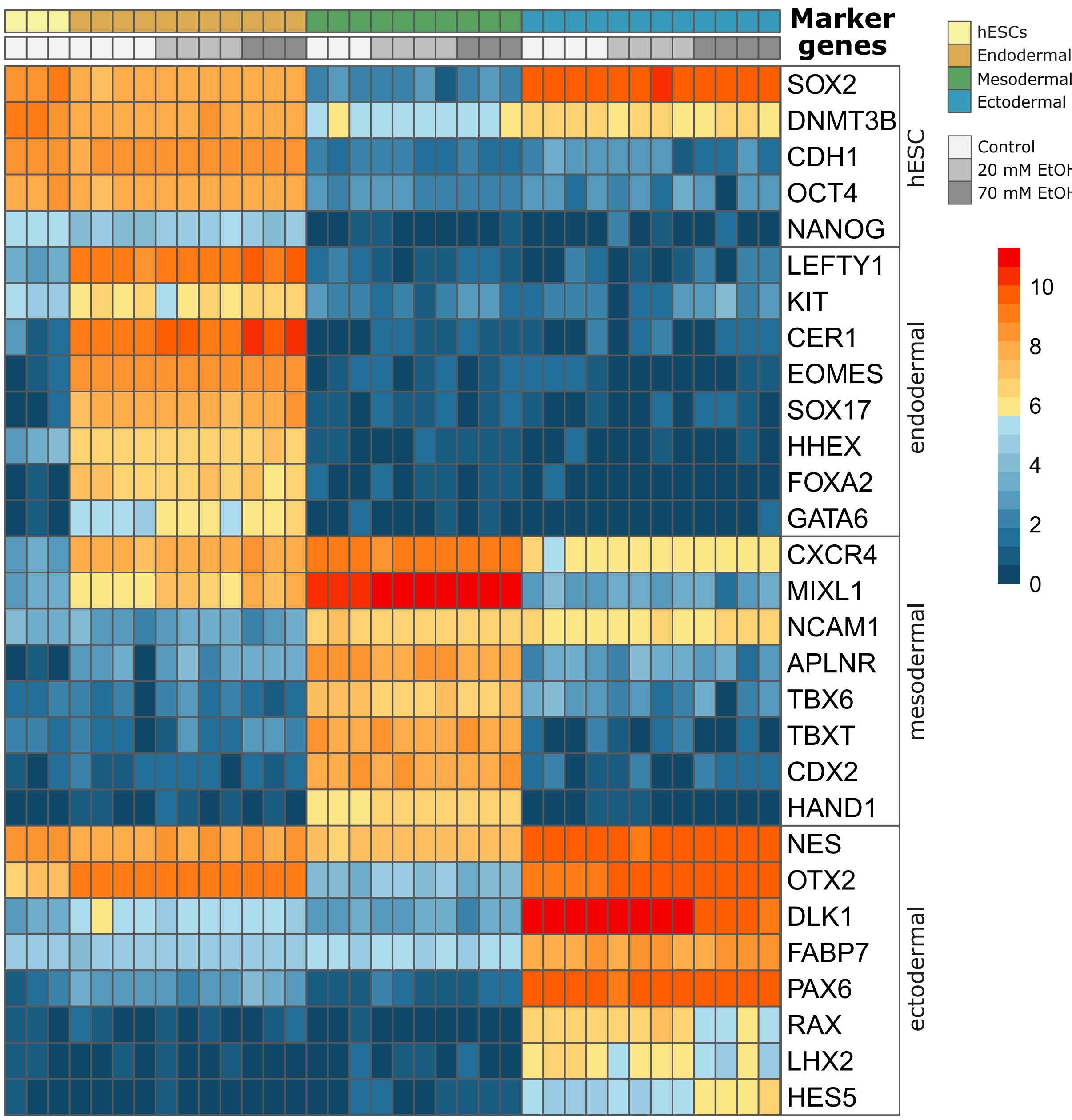

Fig. S4. Validation of hESC differentiation into the germ layer cells by gene expression. Gene expression heatmap of pluripotency and differentiation marker genes in hESCs (control *n* = 3) as well as endodermal (control *n* = 4, 20 mM EtOH *n* = 4, 70 mM EtOH *n* = 3), mesodermal (control *n* = 3, 20 mM EtOH *n* = 4, 70 mM EtOH *n* = 3) and ectodermal cells (control *n* = 4, 20 mM EtOH *n* = 4, 70 mM EtOH *n* = 4).

Disease Models & Mechanisms • Supplementary information

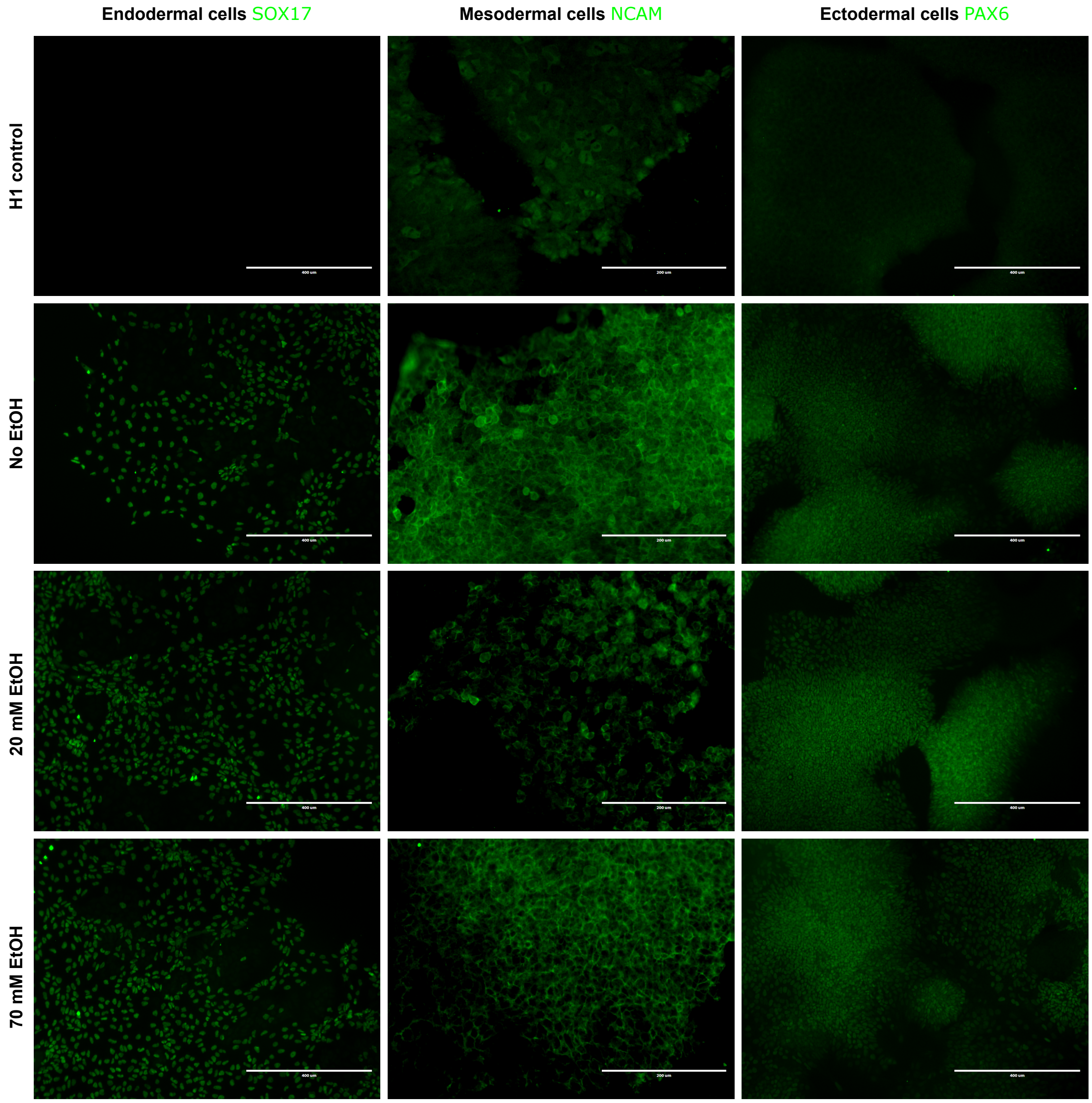

Fig. S5. Validation of hESC differentiation into the germ layer cells by immunofluorescence staining. Immunofluorescence staining of SOX17 in the endodermal cells, NCAM in the mesodermal cells, and PAX6 in the ectodermal cells. Scale bars, 400 μm.

Disease Models & Mechanisms • Supplementary information

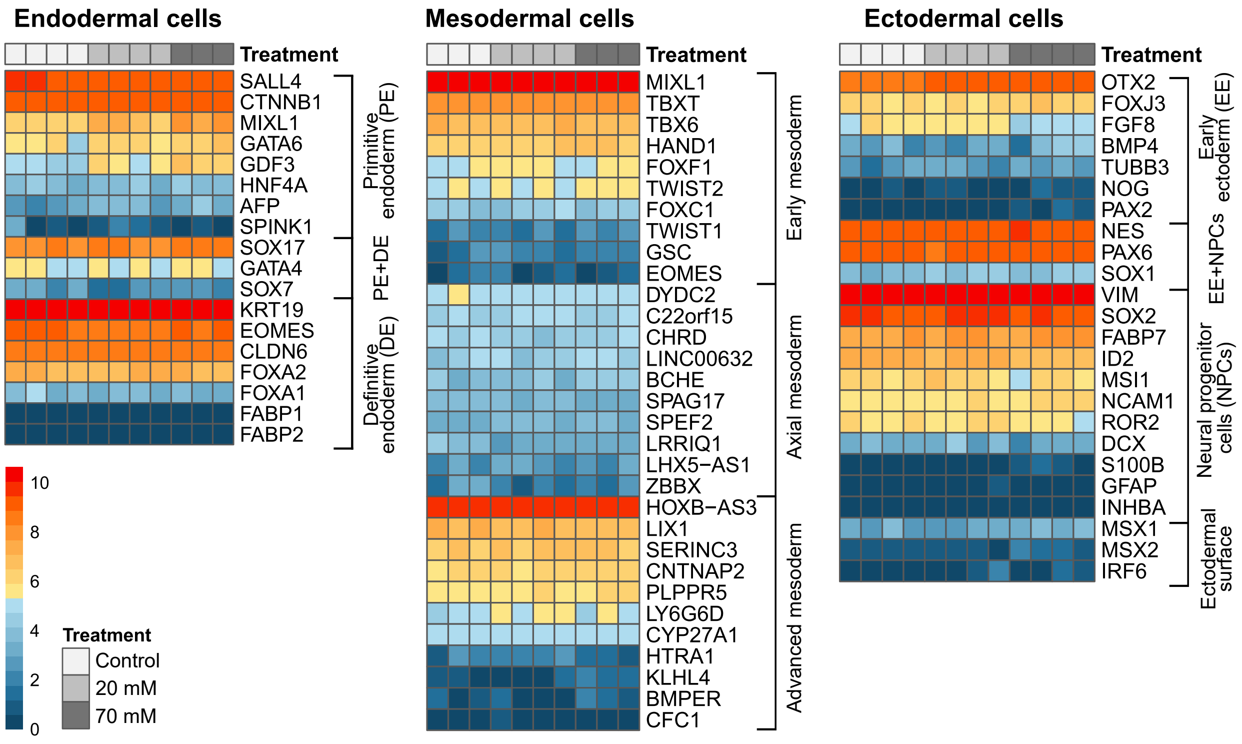

Fig. S6. Examination of potential cell heterogeneity within the germ layers by gene expression. Gene expression heatmap of cell-type-specific marker genes in endodermal (control *n* = 4, 20 mM EtOH *n* = 4, 70 mM EtOH *n* = 3), mesodermal (control *n* = 3, 20 mM EtOH *n* = 4, 70 mM EtOH *n* = 3) and ectodermal cells (control *n* = 4, 20 mM EtOH *n* = 4, 70 mM EtOH *n* = 4). Due to low expression of axial mesodermal marker genes, counts below ten have been excluded from the visualization. EE: early ectoderm, DE: definitive endoderm, PE: primitive endoderm, NPC: neural progenitor cell.

Table S1. EtOH-induced DEGs in the endodermal cells analyzed by mRNA-seq

Available for download at
https://journals.biologists.com/dmm/article-lookup/doi/10.1242/dmm.052150#supplementary-data

Table S2. GO pathway analysis of DEGs in the endodermal cells

Available for download at
https://journals.biologists.com/dmm/article-lookup/doi/10.1242/dmm.052150#supplementary-data

Table S3. EtOH-induced DEGs in the mesodermal cells analyzed by mRNA-seq

Available for download at
https://journals.biologists.com/dmm/article-lookup/doi/10.1242/dmm.052150#supplementary-data

Table S4. EtOH-induced DEGs in the ectodermal cells analyzed by mRNA-seq

Available for download at
https://journals.biologists.com/dmm/article-lookup/doi/10.1242/dmm.052150#supplementary-data

Table S5. GO pathway analysis of DEGs in the ectodermal cell

Available for download at
https://journals.biologists.com/dmm/article-lookup/doi/10.1242/dmm.052150#supplementary-data

Table S6. EtOH-induced DMPs in the endodermal cells analyzed by microarrays

Available for download at
https://journals.biologists.com/dmm/article-lookup/doi/10.1242/dmm.052150#supplementary-data

Table S7. EtOH-induced DMRs in the endodermal cells analyzed by microarrays

Available for download at
https://journals.biologists.com/dmm/article-lookup/doi/10.1242/dmm.052150#supplementary-data

Table S8. GO pathway analysis of endodermal DMPs

Available for download at
https://journals.biologists.com/dmm/article-lookup/doi/10.1242/dmm.052150#supplementary-data

Table S9. EtOH-induced DMPs in the mesodermal cells analyzed by microarrays

Available for download at
https://journals.biologists.com/dmm/article-lookup/doi/10.1242/dmm.052150#supplementary-data

Table S10. EtOH-induced DMRs in the mesodermal cells analyzed by microarrays

Available for download at
https://journals.biologists.com/dmm/article-lookup/doi/10.1242/dmm.052150#supplementary-data

Table S11. EtOH-induced DMPs in the ectodermal cells analyzed by microarrays

Available for download at
https://journals.biologists.com/dmm/article-lookup/doi/10.1242/dmm.052150#supplementary-data

Table S12. EtOH-induced DMRs in the ectodermal cells analyzed by microarrays

Available for download at
https://journals.biologists.com/dmm/article-lookup/doi/10.1242/dmm.052150#supplementary-data

Table S13. GO pathway analysis of ectodermal DMPs

Available for download at
https://journals.biologists.com/dmm/article-lookup/doi/10.1242/dmm.052150#supplementary-data

Table S14. Common genes in the current study and previous early alcohol exposure gene expression and/or DNAm studies

Available for download at
https://journals.biologists.com/dmm/article-lookup/doi/10.1242/dmm.052150#supplementary-data

Table S15. Common genes in the current study and previous DNAm studies of children with FASD

Available for download at
https://journals.biologists.com/dmm/article-lookup/doi/10.1242/dmm.052150#supplementary-data

Table S16. Characteristics, statistical results, and reference spectra for all metabolic features

Available for download at
https://journals.biologists.com/dmm/article-lookup/doi/10.1242/dmm.052150#supplementary-data

Table S17. EtOH-induced significantly altered extracellular metabolites of all germ layer cells.

Available for download at
https://journals.biologists.com/dmm/article-lookup/doi/10.1242/dmm.052150#supplementary-data

Table S18. SMPDB enrichment analysis of the annotated metabolites significantly altered by EtOH.

Available for download at
https://journals.biologists.com/dmm/article-lookup/doi/10.1242/dmm.052150#supplementary-data

Table S19. Correlations between omics analyses

Available for download at
https://journals.biologists.com/dmm/article-lookup/doi/10.1242/dmm.052150#supplementary-data

Supplementary references

Auvinen, P., Vehviläinen, J., Marjonen, H., Modhukur, V., Sokka, J., Wallén, E., Rämö, K., Ahola, L., Salumets, A., Otonkoski, T. et al. (2022). Chromatin modifier developmental pluripotency associated factor 4 (DPPA4) is a candidate gene for alcohol-induced developmental disorders. *BMC Med* 20, 495. https://doi.org/10.1186/s12916-022-02699-1

Bestry, M., Larcombe, A. N., Kresoje, N., Chivers, E. K., Bakker, C., Fitzpatrick, J. P., Elliot, E. J., Craig, J. M., Muggli, E., Halliday, J. et al. (2024). Early moderate prenatal alcohol exposure and maternal diet impact offspring DNA methylation across species. *eLife* 12, RP92135. https://doi.org/10.7554/eLife.92135.3

Boschen, K. E., Ptacek, T. S., Berginski, M. E., Simon, J. M., and Parnell, S. E. (2021). Transcriptomic analyses of gastrulation-stage mouse embryos with differential susceptibility to alcohol. *Dis. Model Mech.* 14, dmm049012. https://doi.org/10.1242/dmm.049012

Cobben, J. M., Krzyzewska, I. M., Venema, A., Mul, A. N., Polstra, A., Postma, A. V., Smigiel, R., Pesz, K., Niklinski, J., Chomczyk, M. A. et al. (2019). DNA methylation abundantly associates with fetal alcohol spectrum disorder and its subphenotypes. *Epigenomics*, 11*,* 767-785. https://doi.org/10.2217/epi-2018-0221

Khalid, O., Kim, J. J., Kim, H. S., Hoang, M., Tu, T. G., Elie, O., Lee, C., Vu, C., Horvath, S., Spigelman, I. et al. (2014). Gene expression signatures affected by alcohol-induced DNA methylomic deregulation in human embryonic stem cells. *Stem Cell Res.* 12, 791-806. https://doi.org/10.1016/j.scr.2014.03.009

Kleiber, M. L., Mantha, K., Stringer, R. L., & Singh, S. M. (2013). Neurodevelopmental alcohol exposure elicits long-term changes to gene expression that alter distinct molecular pathways dependent on timing of exposure. *J. Neurodev. Disord.* 5, 1-19. https://doi.org/10.1186/1866-1955-5-6

Laufer, B. I., Kapalanga, J., Castellani, C. A., Diehl, E. J., Yan, L., and Singh, S. M. (2015). Associative DNA methylation changes in children with prenatal alcohol exposure. *Epigenomics*, 7, 1259-1274. https://doi.org/10.2217/epi.15.60

Legault, L. M., Dupas, T., Breton-Larrivée, M., Filion-Bienvenue, F., Lemieux, A., Langford-Avelar, A., and McGraw, S. (2024). Sex-specific DNA methylation and gene expression changes in mouse

placentas after early preimplantation alcohol exposure. *Environ. Int.* 192, 109014. https://doi.org/10.1016/j.envint.2024.109014

Lussier, A. A., Morin, A. M., MacIsaac, J. L., Salmon, J., Weinberg, J., Reynolds, J. N., Pavlidis, P., Chudley, A. E. and Kobor, M. S. (2018). DNA methylation as a predictor of fetal alcohol spectrum disorder. *Clin Epigenet* 10, 5. https://doi.org/10.1186/s13148-018-0439-6

Portales-Casamar, E., Lussier, A. A., Jones, M. J., MacIsaac, J. L., Edgar, R. D., Mah, S. M., Barhdadi, A., Provost, S., Lemieux-Perreault, L.-P., Cynader, M. S. et al. (2016). DNA methylation signature of human fetal alcohol spectrum disorder. *Epigenetics Chromatin*, 9, 1-20. https://doi.org/10.1186/s13072-016-0074-4

Marjonen, H., Sierra, A., Nyman, A., Rogojin, V., Gröhn, O., Linden, A. M., Hautaniemi, S. and Kaminen-Ahola, N. (2015). Early maternal alcohol consumption alters hippocampal DNA methylation, gene expression and volume in a mouse model. *PloS One*, 10, e0124931. https://doi.org/10.1371/journal.pone.0124931

Disease Models & Mechanisms • Supplementary information
